# Integrated metabolomic and gut microbiota analyses reveal TCA cycle dysfunction in broiler skeletal disorders and the protective effect of L‑malic acid

**DOI:** 10.1186/s40104-026-01498-5

**Published:** 2026-09-13

**Authors:** Yuyang Xue, Min Yao, Xiangjian Peng, Fang Wang, Wei Jing, Fei Zhang, Lulu Mi, Wenju Zhang, Cunxi Nie

**Affiliations:** 1 College of Animal Science and Technology, Bingtuan Key Laboratory for Efficient Utilization of Non-Grain Feed Resources, Shihezi University, Shihezi, 832003 China; 2 School of Medicine, Shihezi University, Shihezi, 832003 China

**Keywords:** Bone health, Broilers, Energy metabolism, Skeletal abnormalities

## Abstract

**Background:**

Skeletal disorders pose a significant challenge to intensive broiler production. However, the metabolic mechanisms underlying the development of skeletal abnormalities in broilers remain unclear. This study aims to reveal the underlying mechanisms through multi-omics analyses and evaluate the ameliorative effects of exogenous L-malic acid supplementation.

**Results:**

The abnormal group exhibited reduced serum concentrations of calcium and phosphorus, as well as decreased bone calcium and phosphorus content. These changes were accompanied by deterioration in bone geometric properties and microstructural parameters. The abnormal group exhibited downregulated expression of osteogenesis-related genes, whereas the mRNA expression levels of osteoclast-related genes were significantly higher than those observed in the normal group (*P* < 0.05). Serum metabolomics revealed that the differentially enriched pathways were primarily associated with energy metabolism, particularly the tricarboxylic acid cycle (TCA cycle). Concurrently, the mRNA expression levels of genes encoding key glycolytic enzymes and key complexes of the electron transport chain were elevated in the abnormal group. The analysis of the gut microbiota revealed an increased abundance of harmful bacteria and a significant decrease in microbes associated with energy metabolism in the abnormal group (*P* < 0.05). Targeted analysis further identified TCA cycle intermediates, fumarate and malate, as robust biomarkers for discriminating skeletal health status. Early dietary supplementation with L-malic acid improved bone health in broilers by increasing TCA activity, as evidenced by enhanced bone mineralization, improved bone microstructural parameters, and an increased OPG/RANKL ratio.

**Conclusions:**

Skeletal abnormalities in broilers are closely associated with gut microbiota dysbiosis accompanied by TCA cycle metabolic disruption. Exogenous L-malic acid supplementation improved bone health by enhancing TCA cycle-related activity, supporting a functional role of TCA cycle-related metabolism in skeletal development.

**Supplementary Information:**

The online version contains supplementary material available at https://doi.org/10.1186/s40104-026-01498-5.

## Background

Ensuring animal health is fundamental to sustainable livestock production. Yellow-feathered broilers are one of the most important poultry breeds for meat production in China, accounting for approximately 50% of the domestic chicken meat market [1]. The Jinling Yellow-feathered broiler is a high-quality commercial breed developed from indigenous Chinese chicken breeds through selective genetic improvement. It is characterized by tender meat, desirable flavor, thin skin and fine bones, with a moderate level of subcutaneous fat deposition and distinct production traits. Currently, genetic improvement of yellow-feathered broilers has increased their body weight from 1 kg to approximately 3 kg and shortened the production cycle, yielding considerable economic benefits [2]. However, the increase in body weight has not been matched by the proportional development of the skeletal system, which disrupt the balance between tissue growth and skeletal development [3] and impairs bone mineralization [4, 5], thereby leading to frequent skeletal disorders in yellow feathered broilers during production.

Concurrently, with the growing demand for chicken meat and the pursuit of higher production efficiency, cage-rearing has become the predominant housing system for broilers, with multi‑tier cage systems being widely adopted. Under cage‑rearing conditions, broilers have limited space for movement, resulting in decreased physical activity [6–8]. These factors have rendered broilers highly susceptible to various skeletal disorders, such as valgus-varus deformities, tibial dyschondroplasia, femoral head necrosis, and epiphyseal plate abnormalities [9, 10]. During slaughter and transportation to slaughterhouses, an immature skeletal structure increases the risk of bone fractures (primarily in the legs and wings) and meat quality defects, ultimately leading to compromised animal welfare and substantial economic losses [11, 12].

The etiology of skeletal abnormalities and disorders in broilers is highly complex, making their definitive classification challenging [13]. However, skeletal growth and homeostasis are governed by the coupled processes of bone formation by osteoblasts and bone resorption by osteoclasts [14, 15]. An imbalance between these processes, particularly when bone resorption exceeds bone formation, can lead to a range of skeletal pathologies including decreased and abnormally elevated bone masses [16]. Most previous studies have aimed to improve bone health through direct dietary supplementation with various functional additives and nutritional interventions. The proposed mechanisms include altering the expression of genes related to bone formation or resorption [17, 18], inhibiting osteoclast activity, enhancing the expression of cartilage matrix-associated genes [19], and modifying the composition of the gut microbiota [20]. Although such interventions have shown promising results, many have failed to target the underlying etiological factors. This lack of targeted interventions may hinder the achievement of optimal outcomes, diminish the efficacy of additives, and ultimately limit their applicability in commercial production.

In this study, we aimed to better understand skeletal disorders in broilers by establishing distinct groups based on comprehensive gait scoring and serum [Ca × P] products, enabling a systematic comparison of phenotypic differences between normal and abnormal bone development. We investigated alterations in gut microbiota composition and metabolomic profiles, with key findings were further validated through targeted metabolomics. Based on the identified key targets, exogenous supplementation was administered to evaluate its efficacy in improving bone health in broilers. This integrated approach allowed us to elucidate the fundamental mechanisms underlying skeletal abnormalities, thereby providing a theoretical foundation for the development of targeted interventions and novel feed additives.

## Materials and methods

### Animals and experimental design

#### Experiment 1

The present study was conducted at Xinjiang Chuangyu Poultry Breeding Co., Ltd. (Shihezi, Xinjiang, China). To better reflect the skeletal health issues of broilers under actual production conditions, Jinling Yellow-feathered male broilers from the same batch and raised under consistent management conditions were selected and housed in a single house. The broilers were provided ad libitum access to feed and water. Management followed conventional commercial broiler rearing procedures: humidity was maintained at 65%, 60%, and 55% during the first, second, and third weeks, respectively, and was maintained at 50% from the fourth week onward. The temperature was set at 34 °C at 1 d of age, decreased by 0.5 °C per day during the first week, and reduced by 0.2 °C per day from the second week until the end of the rearing period. The nutritional levels of the diet were based on the Chinese Agricultural Industry Standard Nutrient Requirements of Yellow-Feathered Broilers (NY/T 3645–2020) [21], divided into starter (d 1–21), grower (d 22–42), and finisher (d 43–63) phases. The formulations and nutrient compositions of the diets for each phase are provided in Supplementary Table S1.

On the day of slaughter (d 70), broilers exhibiting distinctly normal and distinctly abnormal gaits were preliminarily selected, with a total of 60 birds (30 candidates each for the normal group and abnormal groups). Gait was assessed in all candidate birds using the six-point scale described by Knowles et al. [22], with scoring performed independently by a single evaluator. In the present study, gait scores of 0–1 were classified as normal, whereas scores of 3–5 were classified as abnormal. Birds with a score of 2 (intermediate) were excluded to select extreme phenotypes, which allowed for the maximization of between-group differences for subsequent mechanistic analyses.

According to the CLSI‑EP28‑A3c guideline, the mean (*μ*) and standard deviation (SD) of serum calcium-phosphorus [Ca × P] product were calculated from broilers with normal gait (scores 0–1). The lower limit of the normal range was defined as *μ* – 1.5 × SD, and values below this threshold were classified as abnormal.

The grouping criteria were defined as follows: the normal group consisted of broilers with a gait score of 0–1 and a [Ca × P] product not below the threshold; the abnormal group consisted of broilers with a gait score of 3–5 and a [Ca × P] product below the threshold. Blinding was implemented throughout the study process. Ultimately, 15 broilers meeting the criteria for the normal group and 15 meeting the criteria for the abnormal group were randomly selected for downstream analyses.

The chickens were euthanized via intravenous administration of sodium pentobarbital at a dose of 30 mg/kg body weight (BW). Blood was collected from the vein using an 80-mm needle, and serum was separated via centrifugation at 3,000 × *g* for 15 min. The serum was divided into two aliquots: one was stored at −20 °C for analysis of serum Ca, P, and bone metabolic markers, and the other was stored at −80 °C for metabolomics analysis. After slaughter, the tibia and humerus were excised and cleared of surrounding soft tissues. The right tibia and humerus were weighed, and their geometric properties were measured. Subsequently, the proximal portion of the right tibia was fixed in 4% paraformaldehyde solution for micro-computed tomography (micro-CT) scanning and hematoxylin and eosin (H&E) staining. The subchondral cancellous bone of the proximal left tibia was collected into a 2-mL cryotube and stored at −80 °C for subsequent analysis; the remaining portion of the tibia was stored at −20 °C until further analysis. Cecal contents were collected and stored at −80 °C for microbiome analysis.

#### Experiment 2

This study aimed to investigate whether dietary L-malic acid (L-MA) supplementation could improve bone health in broilers. The candidate target identified in this study was based on skeletally normal and abnormal samples collected under conventional production conditions, rather than on a specific disease model, the validation trial of exogenous L-MA supplementation was likewise performed in a conventionally reared broiler flock. Because early growth is a critical period for skeletal development in broilers, the L-MA supplementation trial was conducted during the starter phase (d 1–21) to evaluate its potential to improve bone health.

This study was conducted at Xinjiang Chuangyu Poultry Breeding Co., Ltd. (Shihezi, Xinjiang, China). A total of 480 1-day-old Jinling Yellow-feathered male broilers were randomly divided into four groups, with six replicates per group and 20 birds in each replicate. The experimental period was 21 d. The control (CON) group was fed a basal diet, and the other three treatment groups were fed the basal diet supplemented with 0.05% (LMA), 0.15% (MMA), or 0.3% (HMA) L‑MA.

The feeding and management procedures were identical to those described previously. L-MA was supplied by Huaheng Biotechnology Co., Ltd. (Hefei, China; purity > 99%). On d 21, 10 broilers from each group were selected for slaughter and subsequent sample collection. Serum was separated and stored at −80 °C for targeted metabolomic analysis. The right tibia and humerus were weighed, and their geometric properties were measured. The proximal portion of the right tibia was fixed in 4% paraformaldehyde for micro-CT scanning and histological staining analyses. The subchondral cancellous bone of the proximal left tibia was collected into a 2-mL cryotube and stored at −80 °C; the remaining portion of the tibia was stored at −20 °C until subsequent analysis.

### Leg health assessment

The gait performance of the selected broilers was assessed. Based on the method described by Knowles et al. [22], the gait score was recorded using a six-point scale ranging from 0 (normal gait and sensitive) to 5 (unable to stand or even walk voluntarily).

The assessment of bone metabolism inevitably involves evaluating serum Ca and P levels, as serum biochemical parameters are indicative of systemic physiological status [23]. Previous studies have emphasized the regulatory role of serum Ca, P, and their product ([Ca × P]) in bone mineralization and skeletal homeostasis [24].

Therefore, following the initial screening via gait scoring, broilers were further categorized according to the serum [Ca × P] product into normal (N) and abnormal (A) groups. Fifteen samples were selected from each group for downstream analyses. The results indicated that abnormal serum [Ca × P] product levels were closely associated with abnormal gait scores (Table 1).

**Table 1:** Leg health assessment and serum Ca × P product in normal and abnormal groups

Item	Number	Gait score	[Ca × P] product, mg^2^/dL^2^	Item	Number	Gait score	[Ca × P] product, mg^2^/dL^2^
Normal	1	0	46.08	Abnormal	1	5	31.49
	2	0	42.29		2	5	30.84
	3	0	43.80		3	4	32.55
	4	0	48.63		4	5	28.12
	5	0	45.25		5	4	30.93
	6	0	48.35		6	5	32.40
	7	0	49.19		7	5	31.82
	8	0	44.08		8	5	30.22
	9	0	58.38		9	5	30.40
	10	1	50.14		10	5	26.40
	11	0	40.08		11	4	33.75
	12	0	41.21		12	5	34.88
	13	0	41.52		13	5	33.49
	14	1	41.03		14	5	32.79
	15	0	43.12		15	5	33.16

Gait score 0: bird walked normally with no detectable abnormality; Gait score 1: bird had a slight defect that was difficult to precisely define; Gait score 2: bird had definite and identifiable defect in gait; Gait score 3: bird had obvious gait defect that affected movement ability; Gait score 4: bird had severe gait defect; Gait score 5: bird was incapable of sustained walking

### Bone measurement and geometrical characteristics

After the adherent soft tissues from the tibia and humerus were removed, the bones were weighed. Bone length, proximal and distal widths, as well as the external (H) and internal (h) diameters in the mediolateral plane and the external (B) and internal (b) diameters in the anteroposterior plane at the mid-diaphyseal transverse section were measured using a digital vernier caliper (precision ± 0.01 mm; Ruineng NR0158, Ningbo Yuanneng Trading Co., Ltd., Ningbo, China). Following previously established methods, the following geometric properties were calculated based on these measurements: MRWT, CSA, CI, WTv, CIv, and CSMI [25, 26]. All analyses were performed by the same investigator.

### Calcium phosphorus metabolism and bone metabolism markers

Serum Ca and P concentrations were determined using commercial assay kits (Nanjing Jiancheng Bioengineering Institute and Jiangsu Aidisheng Biotechnology Co., Ltd., respectively), according to the manufacturers’ instructions. The serum concentrations of bone metabolic markers, including PINP, COL1A1, β‑CTX, BALP, and TRAP, were measured using corresponding commercial enzyme-linked immunosorbent assay kits (Shanghai Enzyme-linked Biotechnology Co., Ltd.). These markers are well-established in bone metabolism research. BALP, COL1A1 and PINP are established bone formation markers, while TRAP and β-CTX are established bone resorption markers [27, 28].

Tibial bone tissue samples were thawed at 4 °C, dried at 105 °C, and defatted in petroleum ether for 48 h, with the petroleum ether being replaced every 24 h. The samples were subsequently dried in an oven at 105 °C to a constant weight. The weight of the defatted bone was determined by weighing. Ash content was determined using a muffle furnace method. The detection of Ca and P in bone was carried out according to the Chinese national standards GB/T 6436–2018 [29] and GB/T 6437–2018 [30], respectively.

Bone tissue samples were pulverized into a fine powder under liquid nitrogen. The ATP and reactive oxygen species (ROS) levels in bone tissue were measured using commercial assay kits (batch No. G4309 and G1746, Wuhan Servicebio Biotechnology Co., Ltd.) via chemiluminescence and fluorescence detection, respectively. The fluorescence intensity was recorded using a multifunction microplate reader (SpectraMax Mini, Shanghai, China).

### Bone microstructural parameter, bone histomorphometry, and immunofluorescence analyses

A segment of the tibia extending from the mid-diaphyseal region to the proximal end was fixed in 4% paraformaldehyde and scanned using a NEMO micro-CT system (NMC-200; Pingsheng Medical Technology, Wuhan, China) for three-dimensional reconstruction and quantitative data analysis. Micro‑CT scanning and analysis were performed according to established guidelines for bone microstructure assessment and relevant literature, with modifications tailored to the experimental animals used in this study [31, 32]. The scanning parameters were set as follows: tube voltage, 80 kV; tube current 0.06 mA, source-to-detector distance, 410 mm; source-to-object distance, 90 mm; acquisition frame rate, 20 frames/s; reconstruction type, FDK; and CT threshold, 453.8. Following micro-CT scanning, the fixed proximal tibial segments were decalcified in 10% ethylenediaminetetraacetic acid solution (pH 7.4), embedded in paraffin, and sectioned for H&E staining [33, 34].

Masson staining and immunofluorescence staining of COL1A1 and OCN were performed in the L‑MA supplementation experiment. Antibodies against COL1A1 and OCN were purchased from Proteintech (batch No. 67288‑1 and 23418‑1‑AP, Wuhan, China). The immunofluorescence protocol was adapted and optimized for bone tissue [35]. Sections were fixed with 4% formaldehyde for 30 min and permeabilized with 0.1% Triton X‑100 for 10 min. After being briefly dried, the sections were encircled with a hydrophobic pen, blocked with 3% BSA for 30 min, and incubated overnight at 4 °C with diluted primary antibodies in a humidified chamber. The sections were washed three times (5 min each) and incubated with secondary antibodies (Servicebio, China) for 50 min at room temperature in the dark. Nuclei were counterstained with DAPI. Fluorescence intensity of COL1A1 and OCN was analyzed using Saiviewer software (Wuhan, China). For each section, 15 randomly selected fields were captured, and three sections were analyzed for each treatment group.

### RNA extraction and quantitative real-time PCR

The mRNA expression of bone metabolism-related genes were analyzed using quantitative real-time PCR (qPCR). Total RNA was extracted from bone tissue using the Easy Pure RNA Kit and subsequently reverse-transcribed into first-strand cDNA using the Easy Script First-Strand cDNA Synthesis Super Mix kit (both from TransGen Biotech Co., Ltd., Beijing, China) according to the manufacturer’s protocols. Each reverse transcription reaction contained 1.5 μg of total RNA. qPCR was performed using a real-time PCR detection system (Hangzhou Bioer Technology Co., Ltd., Hangzhou, China). Primer design information is shown in Table S2. All reactions were performed in triplicate (technical replicates). The mRNA level of the target gene was calculated using the 2^−ΔΔCt^ method [36].

### Serum metabolomic analysis

Metabolomic analyses were performed according to the manufacturer’s protocol (Panomix, Suzhou, Jiangsu, China). Serum sample preparation was performed following the protocol of Demurtas et al. [37]. Briefly, 50 μL of serum sample was mixed with 200 μL of methanol containing an internal standard (2-amino-3-(2-chloro-phenyl)-propionic acid) at 4 °C and incubated for 15 min for protein precipitation. After vortexing and centrifugation at 21,912 × *g* and 4 °C, the supernatant was collected, filtered through a 0.22-μm membrane, and transferred to a vial for Liquid Chromatography-Mass Spectrometry (LC–MS) analysis. The chromatographic conditions and spectrometry parameters were based on the protocols described by Zelena et al. [38] and Want et al. [39], respectively, and are specified as follows. Chromatographic separation was achieved using an ultra-performance liquid chromatography system (Thermo Fisher Scientific, USA) equipped with an ACQUITY UPLC HSS T3 column (2.1 mm × 100 mm, 1.8 µm; Waters, Milford, MA, USA). The separation was conducted at a flow rate of 0.3 mL/min and column temperature of 40 °C, with an injection volume of 2 μL. The metabolites were detected using a Thermo Orbitrap Exploris 120 mass spectrometer (Thermo Fisher Scientific, USA) equipped with an electrospray ionization (ESI) source. Data were acquired in both the positive and negative ionization modes.

### 16S rRNA sequencing of cecal microbiota

16S rRNA gene sequencing was conducted according to the manufacturer’s protocol (Panomix Biomedical Technology Co., Ltd., Suzhou, China). PCR amplification was performed using TransGen Biotech’s Pfu high-fidelity DNA polymerase, with a strictly controlled number of amplification cycles. Purified PCR products were quantified fluorometrically using the Quant-iT PicoGreen dsDNA Assay Kit (Invitrogen, Thermo Fisher Scientific, USA) and a Microplate reader (FLx800, BioTek, Winooski, VT, USA). Sequencing libraries were prepared using the Illumina TruSeq Nano DNA LT Library Prep Kit. The generated raw FASTQ files were subjected to quality filtering. Raw data were denoised using the DADA2 algorithm, and the resulting representative dereplicated sequences are generally referred to as amplicon sequence variants (ASVs) [40]. Taxonomic annotation of each ASV representative sequence was conducted using the classify‑sklearn machine learning algorithm in QIIME2, with a pre‑trained Naïve Bayes classifier based on the relevant database and using default parameters [41]. The taxonomic annotation of the sequenced 16S rRNA genes was performed using the Greengenes database (Release 13.8, http://greengenes.secondgenome.com/) [42]. Principal coordinate analysis (PCoA) based on Bray–Curtis distances was performed to visualize differences in microbial community structure, and statistical significance was assessed using the *Adonis* function in the *vegan* package.

### Targeted metabolomic validation

Targeted metabolomic analysis was conducted according to the manufacturer’s protocol (Panomix Biomedical Technology Co., Ltd., Suzhou, China), using a commercial energy metabolism kit (Glucose metabolism-related, covering 41 metabolites). An appropriate amount of the seven internal standards for energy metabolism was weighed, 1 mg/mL of internal standard single-label reserve solution was prepared with water and methanol, an appropriate amount of each single-label reserve solution was measured and diluted with 50% acetonitrile/water solution to produce a mixed internal standard reserve solution, which was stored at −20 °C until needed. To prepare a standard solution, 80 µL of secondary working solution and 20 µL of mixed internal standard reserve solution were mixed in an injection vial. Metabolites were extracted following Aretz et al. [43]. Chromatographic separation was performed on an ExionLC ultra‑performance liquid chromatography system (AB Sciex, USA) using an Agilent Eclipse XDB‑C18 column (Agilent, USA), following the method described by Sullivan et al. [44]. Mass spectrometric conditions were established according to the protocol of Bi et al. [45], with a Luna^®^ 3 μm NH_2_ column (Phenomenex) employed for separation. Quantitative analysis of all samples was conducted based on the metabolite calibration curves.

The targeted metabolomics analysis in the L-MA supplementation trial was conducted by Sanshu Biotechnology Co., Ltd. (Suzhou, Jiangsu, China) (Energy metabolism-related, covering 100 metabolites). Samples were thawed slowly at 4 °C, then mixed with pre-chilled methanol/acetonitrile solution (1:1, v/v) and 25 μL of internal standard, followed by vortex mixing. The mixture was sonicated at low temperature for 5 min, incubated at −20 °C for 1 h to precipitate proteins, and centrifuged at 21,912 × *g* and 4 °C for 20 min. The reaction was incubated with shaking at room temperature for 60 min. Then, 100 μL of pre-chilled 50% methanol solution was added, the mixture was vortexed and centrifuged at 21,912 × *g* and 4 °C for 15 min, and the supernatant was injected for analysis. Mixed standard stock solutions were prepared using individual reference standards, and a series of calibration solutions were obtained by serial gradient dilution. Each calibration level was processed following the same sample preparation procedure. Sample separation was performed on an LC-30AD ultra-high performance liquid chromatography system (Shimadzu, Japan) using Amide and C18 chromatographic columns, respectively. Mass spectrometric analysis was performed using a 6500+ QTRAP mass spectrometer (SCIEX, Framingham, MA, USA) in negative ion mode. Multiple reaction monitoring (MRM) mode was used for detection.

### Data analysis

All data were first assessed for normality using the Shapiro–Wilk test in SAS 9.4 (SAS Inc., Cary, NC, USA). Independent sample *t*-tests were used to analyze the differences between the two groups. Data are presented as mean and SEM (standard error of the mean). Differences were considered significant at *P* < 0.05, highly significant at *P* < 0.01, and extremely significant at *P* < 0.001. Unless otherwise specified, all graphs were generated using GraphPad Prism 9.0 (San Diego, CA, USA).

Power analysis was conducted using G*Power version 3.1 software [46, 47]. The effect size Cohen’s d was estimated as 3.88 based on the serum [Ca × P] product data. Setting the significance level at α = 0.05 and the desired statistical power at 1 − β = 0.85, the calculation indicated a minimum sample size of four broilers per group. Accordingly, 10 broilers per group were used for the subsequent sequencing analyses in this study.

The raw mass spectrometry data from untargeted metabolomics were converted to mzXML format using the MSConvert tool in the Proteowizard software package (v3.0.8789). Peak detection, filtering, and alignment were carried out using the XCMS package in R software, with the following parameter settings: bw = 2, ppm = 15, peakwidth = c (5, 30), mzwid = 0.015, mzdiff = 0.01, and method = “centWave” [48]. Following the generation of the quantitative feature list, total peak area normalization was employed to correct the data and minimize systematic errors. For multiple comparisons in metabolomics data, we applied the Benjamini–Hochberg false discovery rate (FDR) correction. Orthogonal partial least‑squares discriminant analysis (OPLS‑DA) was used to visualize the differences between groups (Fig. S1, Additional file 1) [49].

By integrating the observed skeletal phenotypic differences, OPLS-DA was also performed based on gait scores, serum [Ca × P] products, and tibial geometric properties. To investigate alterations in metabolites and metabolic pathways between the normal and abnormal groups, the Mann–Whitney U test was employed to identify differentially abundant serum metabolites, as most annotated metabolites did not follow a normal distribution. Quantitative metabolic pathway enrichment analysis was performed using the Kyoto Encyclopedia of Genes and Genomes (KEGG) database.

For gut microbiota analysis, β-diversity was visualized using PCoA plots based on Bray–Curtis distances. Differential microbial taxa were identified using linear discriminant analysis effect size (LEfSe) [50]. Functional prediction of the microbial community was performed using PICRUSt2 software (version 2.6.0) against the MetaCyc database [51]. Correlation analyses between different parameters were performed using the Spearman’s rank correlation method. Six machine learning models—K-Nearest Neighbor, Random Forest, Support Vector Machine, Gaussian Naive Bayes, Logistic Regression, and Decision Tree—were employed to evaluate and compare biomarkers classification performance [52]. Receiver operating characteristic (ROC) analysis was performed to evaluate biomarker panels [53]. In the present study, the ROC analyses were performed using the model training dataset. To evaluate the robustness of the classification models and to mitigate the risk of overfitting, we adopted 10-fold stratified cross‑validation.

In the L-MA supplementation experiment, all data were first checked for normality (Shapiro–Wilk test) and homoscedasticity (Levene test). One-way analysis of variance (ANOVA) was conducted to analyze the significance of differences between treatment groups. Independent sample *t*-tests were used to analyze the differences between the two groups. Linear and quadratic regression analyses were performed on the different treatment groups to test the linear and quadratic effects of the supplementation levels on different outcomes.

## Results

### Skeletal phenotypic characteristics analysis between normal and abnormal groups

#### Ca and P metabolism

**Fig. 1:**
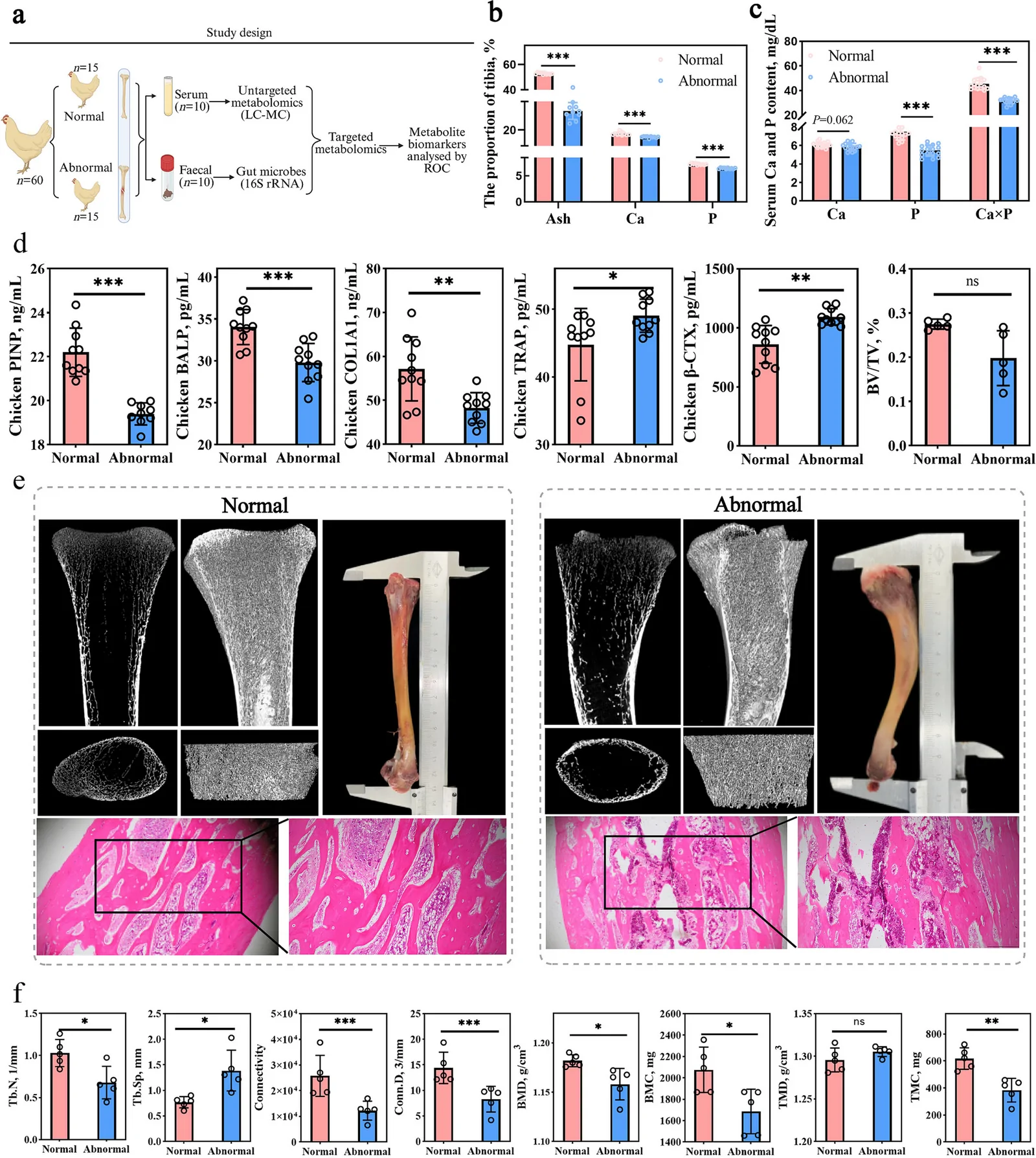
Phenotypic differences in bone between normal and abnormal groups. **a** Schematic diagram of study design (Created in Biorender, https://BioRender.com/yrzivpx). **b** Bone Ca, P, and ash content in the tibia. **c** Serum Ca, P levels, and Ca × P product. **d** Serum levels of bone metabolic markers. **e** Representative micro-CT scanning images (Scale bar, 10 mm) and H&E staining of proximal-midshaft tibia. **f** Corresponding trabecular morphometric analysis. Tb.N, Tb.Sp, connectivity, Conn.D, BMD, BMC, TMD, and TMC represent the number of trabeculae, trabecular separation, connectivity, and connective density, bone mineral density, bone mineral content, tissue mineral density, and tissue mineral content, respectively (Scale bar, 200 μm). ^*^P < 0.05; ^**^P < 0.01; ^***^P < 0.001; ns, not significant

Analysis of Ca and P contents in serum and bone revealed a significant difference between the normal and abnormal groups (*P* < 0.05; Fig. 1b and c). Furthermore, as tibial ash content and inorganic mineral levels were closely associated with bone quality, significantly lower bone Ca, P, and ash contents (*P* < 0.05) were observed in the abnormal group than in the normal group, suggesting compromised bone calcification in a subset of broilers under commercial cage-rearing conditions.

#### Serum bone metabolic markers

Serum levels of bone resorption and formation markers were determined using enzyme-linked immunosorbent assay kits (Fig. 1d). The results showed that TRAP and CTSK contents were significantly higher in the abnormal group than in the normal group (*P* < 0.05). Conversely, the concentrations of the bone formation markers PINP, COL1A1, BALP were significantly reduced in the abnormal group (*P* < 0.05).

#### Bone microstructural parameters

The proximal midshaft of the tibia was subjected to micro-CT and H&E staining to observe microstructural changes in the bone tissue. The results revealed a low bone mass phenotype in the abnormal group, which was characterized by an enlarged medullary cavity, sparse trabeculae, and impaired connectivity (Fig. 1e). These observations were corroborated through morphometric analyses of the trabecular indices (Fig. 1f), including trabecular number (Tb.N), trabecular separation (Tb.Sp), connectivity, and connectivity density (Conn.D), bone mineral density (BMD), bone mineral content (BMC), and tissue mineral content (TMC) were significant differences between the normal and abnormal groups (*P* < 0.05). Collectively, these findings indicate bone loss in broilers under cage-rearing conditions.

#### Bone measurement and geometrical characteristics

Analysis of basic bone indices and geometric properties, which are key metrics for assessing skeletal health, revealed significant differences between the two groups in both the humerus and tibia. The abnormal group exhibited marked reductions in tibial weight, bone length, proximal width, mean relative wall thickness (MRWT), vertical wall thickness (WTv), vertical cortical index (CIv), cross-sectional moment of inertia (CSMI), and cortical cross-sectional area (CSA) compared with the normal group (*P* < 0.05) (Table 2). Similar trends were observed in the humerus, where the abnormal group showed significantly lower MRWT, WTv, CIv, and CSA (*P* < 0.05).

**Table 2:** Bone geometric parameters of the tibia and humerus in normal and abnormal groups

Items			Normal	Abnormal	SEM	*P*-value
Tibia	Weight, g		29.69	24.85	1.03	< 0.001
	Length, mm		133.28	128.27	2.19	0.030
	Pew, mm		32.31	29.70	0.63	< 0.001
	Dew, mm		21.47	20.50	0.49	0.056
	M-L	Hh, mm	10.66	9.31	0.35	0.001
		hh, mm	7.19	6.62	0.34	0.102
	A-P	Hv, mm	9.96	8.04	0.49	0.001
		hv, mm	6.38	5.85	0.276	0.069
	MRWT		0.53	0.41	0.06	0.028
	WTv, mm		1.79	1.09	0.21	0.002
	CI		0.32	0.29	0.03	0.217
	CIv		0.35	0.27	0.03	0.014
	CSMI, mm^4^		477.74	180.11	95.13	0.004
	CSA, mm^2^		47.86	28.38	4.72	< 0.001
Humerus	Weight, g		13.68	12.52	0.57	0.051
	Length, mm		89.96	87.90	1.10	0.073
	Pew, mm		26.18	26.35	0.84	0.834
	Dew, mm		24.71	21.74	1.98	0.144
	M-L	Hh, mm	9.88	8.17	0.39	< 0.001
		hh, mm	6.31	5.67	0.50	0.212
	A-P	Hv, mm	8.08	6.78	0.46	< 0.001
		hv, mm	4.89	4.81	0.30	0.785
	MRWT		0.65	0.44	0.06	0.004
	WTv, mm		1.59	0.99	0.19	0.003
	CI		0.37	0.30	0.03	0.067
	CIv		0.39	0.29	0.04	0.007
	CSMI, mm^4^		279.59	96.84	98.55	0.074
	CSA, mm^2^		39.20	22.12	4.36	0.001

*Hh* Horizontal outer diameter, *hh* Horizontal inner diameter, *Hv* Vertical outer diameter, *hv* Vertical inner diameter, *M-L* Mediolateral direction, *A-P* Anteroposterior direction, *PEW* Proximal epiphysis width, *DEW* Distal epiphysis width, *MRWT* Mean relative wall thickness, *WTv* Vertical wall thickness, *CI* Cortical index, *CIv* Vertical Cortical Index, *CSMI* Moment of inertia, *CSA* Cortical cross-sectional area. The following table is the same

### Serum metabolomic analysis between normal and abnormal groups

**Fig. 2:**
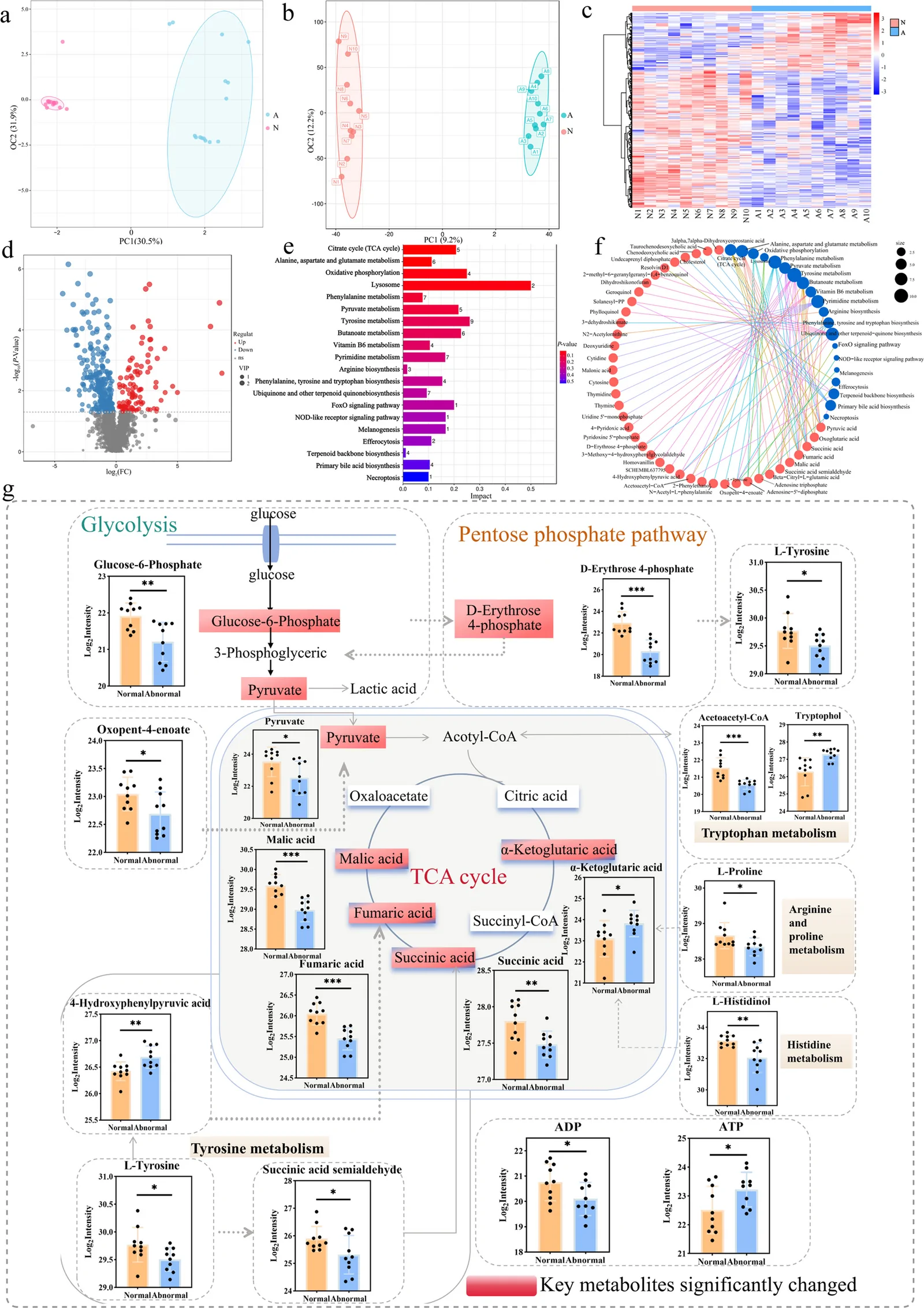
Serum metabolic signatures in normal and abnormal groups. **a** OPLS-DA score plot based on gait score, serum Ca × P product, and tibial geometric characteristics. **b** OPLS-DA of metabolic profiles. **c** Heatmap analysis. **d** Volcano plot of metabolites. **e** KEGG enrichment analysis. **f** Networks of metabolic pathways and metabolites. **g** Alterations in serum metabolic pathways. ^*^P < 0.05; ^**^P < 0.01; ^***^P < 0.001; ns, not significant

The two groups showed distinct separation in their skeletal phenotypes (Fig. 2a). Similarly, the serum metabolomic profiles analyzed using OPLS-DA revealed a clear separation between the normal and abnormal groups (Fig. 2b). Cluster analysis indicated that the differentially abundant metabolites could be distinctly segregated between the two groups (Fig. 2c). A volcano plot was used for binary comparison of the overall metabolites between the two groups, illustrating the differences in individual metabolites (Fig. 2d). KEGG pathway analysis identified 68 enriched metabolic pathways. Four pathways showed significant alterations between the normal and abnormal groups, including the tricarboxylic acid (TCA) cycle; alanine, aspartate, and glutamate metabolism; oxidative phosphorylation (OXPHOS); and lysosome pathways (*P* < 0.05; Fig. 2e). Furthermore, significant disparities were observed in the amino acid metabolism, particularly in pathways involving tyrosine, tryptophan, proline, and histidine. These amino acids and their derivatives were enriched in associated pathways and were ultimately catabolized into TCA cycle intermediates (Fig. 2f and g).

### mRNA expression and mitochondrial function analysis between normal and abnormal groups

Based on the observed skeletal phenotypic differences, we analyzed serum metabolites between the two groups. The results indicated that the enriched metabolic pathways were primarily associated with energy metabolism, including various amino acid metabolic pathways and the TCA cycle. Consequently, we examined the mRNA expression levels of key enzymes involved in glycolysis and OXPHOS as well as key regulatory genes involved in bone formation and resorption. The results demonstrated that the mRNA expression levels of the bone resorption-related genes *RANKL*,* MMP9*,* NFATC1*,* CTSK*,* TRAP*, and *ATP6V0D2* were significantly higher in the abnormal group than in the normal group (*P* < 0.05). Conversely, the expression of the bone formation-related genes *OCN*,* OPN*,* ALPL*, and *RUNX2* was significantly lower in the abnormal group (*P* < 0.05). Although the mRNA levels of *COL1A1* were lower in the abnormal group, the difference was not statistically significant (*P* > 0.05).

**Fig. 3:**
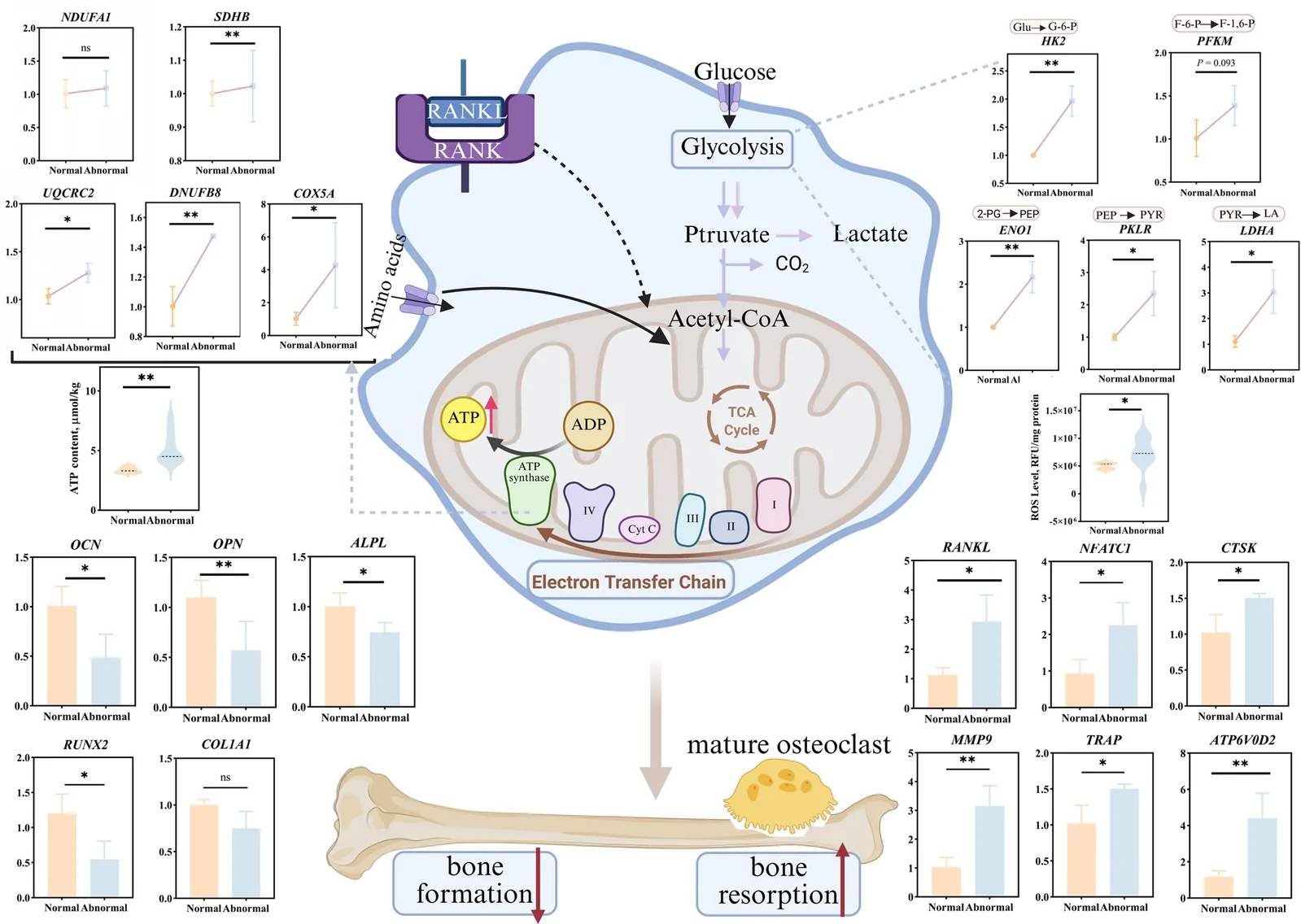
Analysis of mRNA expression of bone metabolism-related genes and functional parameters (ATP and ROS levels) in bone tissue of the normal and abnormal groups. ^*^P < 0.05; ^**^P < 0.01; ns, not significant. Some materials were created in Biorender (https://BioRender.com/44mp4l0)

The mRNA expression levels of key glycolytic enzymes, including *HK2*, *PFKM*, *PKLR*, *LDHA*, and *ENO1*, were significantly higher in the abnormal group than in the normal group (*P* < 0.05). Accordingly, we examined the expression of genes encoding key subunits of mitochondrial electron transport chain (ETC) complexes. The mRNA levels of *SDHB*, *NDUFB8*, *UQCRC2*, and *COX5A* were significantly higher in the abnormal group than in the normal group (*P* < 0.05; Fig. 3).

Compared with the normal group, the levels of ATP and ROS in bone tissue were significantly elevated in the abnormal group (*P* < 0.05; Fig. 3).

### Gut microbial composition between normal and abnormal groups

**Fig. 4:**
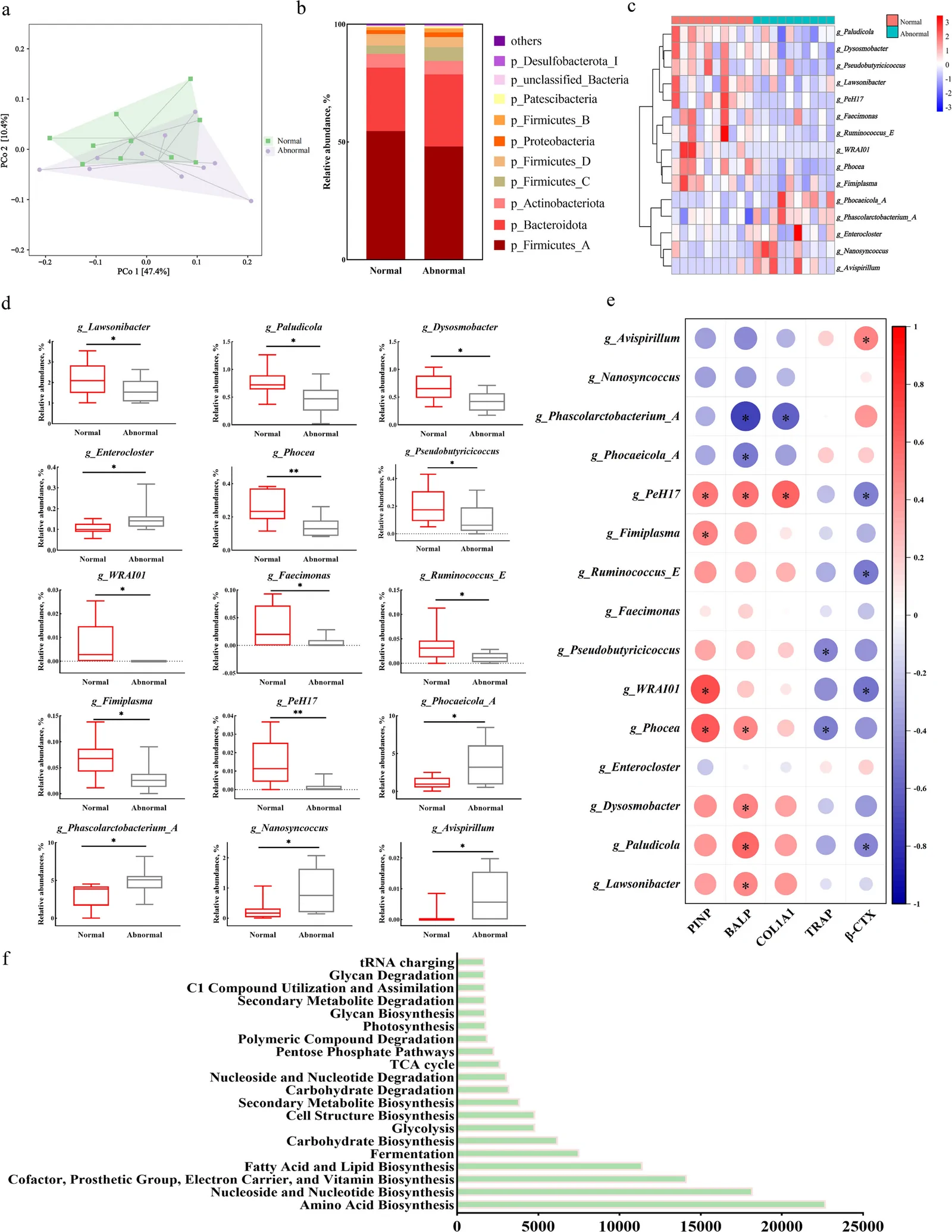
Gut microbiota composition in normal and abnormal groups. **a** Principal coordinate analysis (PCoA) between the normal and abnormal groups. **b** Composition analysis at the phylum level. **c** Heatmap of differentially abundant microbiota at the genus level. **d** LEfSe analysis screened out the genus-level microorganisms in the normal and abnormal groups. **e** Spearman correlation analysis between differential microbial genera and serum bone metabolic markers. **f** Relative abundance of MetaCyc functional pathways. ^*^P < 0.05; ^**^P < 0.01

Analysis of the cecal microbiota composition via 16S rRNA gene sequencing revealed distinct differences between the normal and abnormal groups. PCoA based on the Bray–Curtis dissimilarity confirmed a clear separation between the two groups. The first principal coordinate (PCo1) accounted for 47.4% of the total variance, whereas the second principal coordinate (PCo2) explained 10.4% (Fig. 4a). At the phylum level, Firmicutes_A was the predominant taxon, followed by Bacteroidota, Actinobacteriota, Firmicutes_C, and Firmicutes_D. The abnormal group showed a 6.56% decrease in the abundance of Firmicutes_A and a 3.68% increase in the abundance of Bacteroidota compared with the normal group, but the difference was not statistically significant (*P* > 0.05). In contrast, the abundance of Firmicutes_C and Patescibacteria were increased to varying degrees in the abnormal group (Fig. 4b, Supplementary Table S3).

Heatmap analysis at the genus level revealed significant differences in microbial abundance between the two groups. The abundances of *Phocaeicola_A*, *Phascolarctobacterium_A*, *Nanosyncoccus*, and *Avispirillum* were significantly higher in the abnormal group than in the normal group (*P* < 0.05). Conversely, the abnormal group exhibited a significantly lower abundance of *Paludicola*, *Dysosmobacter*, *Pseudobutyricicoccus*, *Lawsonibacter*, *PeH17*, *Faecimonas*, *Ruminococcus_E*, *WRAI01*, *Phocea*, and *Fimiplasma* (*P* < 0.05; Fig. 4c and Fig. S1b).

Functional prediction identified the three most abundant functional pathways: Amino Acid Biosynthesis, Nucleoside and Nucleotide Biosynthesis, and Cofactor, Prosthetic Group, Electron Carrier, and Vitamin Biosynthesis (Fig. 4f). Spearman correlation analysis indicated significant associations between the gut microbiota and serum bone metabolic markers. PINP concentrations were positively correlated with *WRAI01*, *Phocea*, *PeH17*, and *Fimiplasma* (*P* < 0.05). BALP concentrations were positively correlated with *Lawsonibacter*, *Paludicola*, *Dysosmobacter*, *Phocea*, and *PeH17*, but negatively correlated with *Phocaeicola_A* and *Phascolarctobacterium_A*. COL1A1 concentrations were positively correlated with *PeH17* but negatively correlated with *Phascolarctobacterium_A*. TRAP concentrations were negatively correlated with *Phocea* and *Pseudobutyricicoccus*, and β-CTX concentrations were negatively correlated with *Paludicola*, *WRAI01*, *Ruminococcus_E*, and *PeH17* but positively correlated with *Avispirillum* (*P* < 0.05; Fig. 4e).

### Correlation analysis

**Fig. 5:**
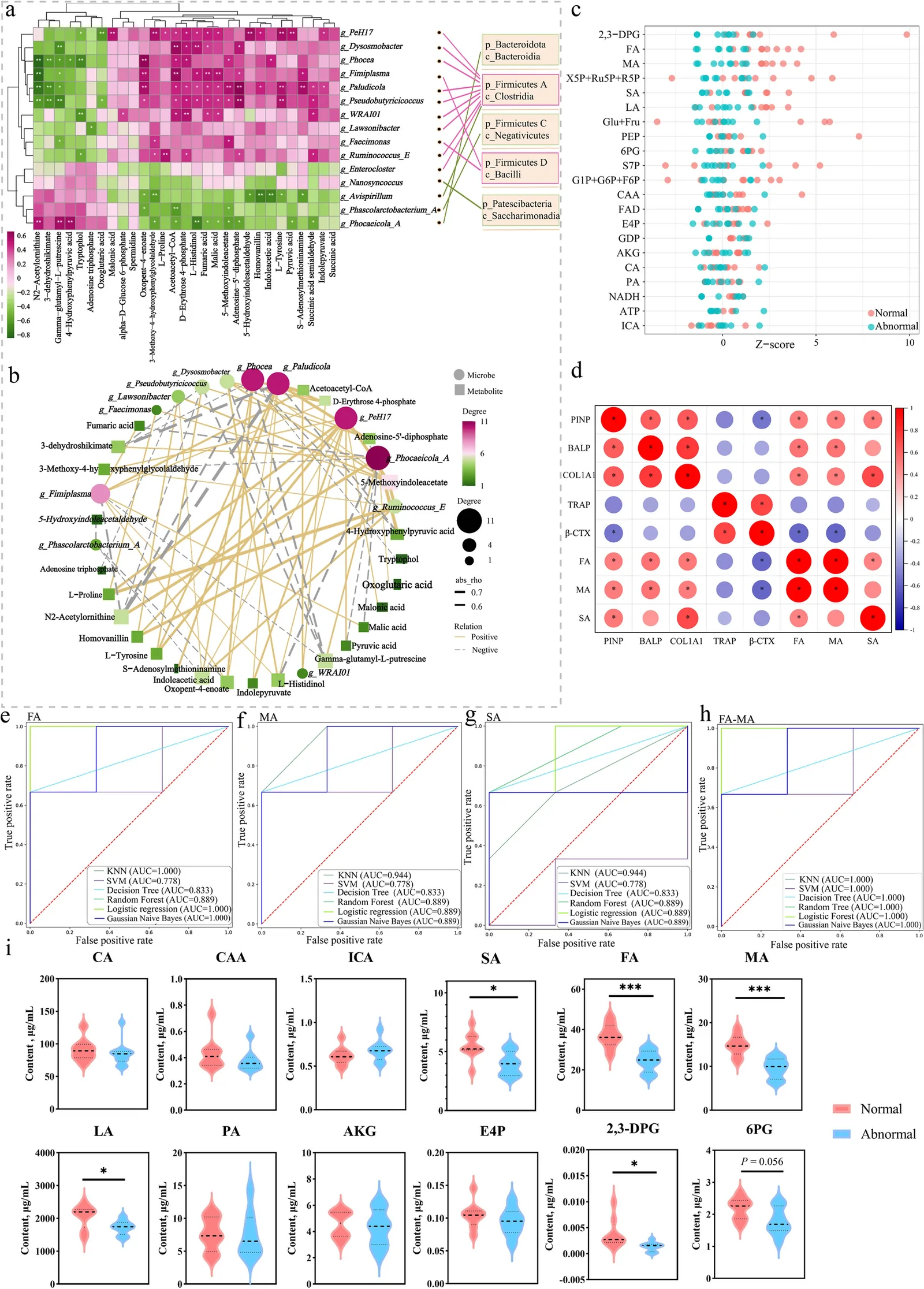
Correlation analysis and ROC analysis. **a** Heatmap of correlations between key metabolites and differential microbial genera. **b** Correlation network diagram filtered based on *P* < 0.05 and |*r*| > 0.5. **c** Z-score plot of targeted characteristic metabolites. **d** Correlation analysis of FA, MA, and SA with serum bone metabolic markers. **e** ROC analysis of FA based on six machine learning methods. **f** ROC analysis of MA based on six machine learning methods. **g** ROC analysis of SA based on six machine learning methods. **h** ROC analysis of the FA and MA combination based on six machine learning methods. ROC: Receiver operating characteristic. **i** Targeted metabolomics analysis of TCA cycle and glycolysis‑related metabolites. ^*^P < 0.05; ^**^P < 0.01; ^***^P < 0.001

To further investigate the association between gut microbiota and serum metabolites related to skeletal abnormalities, Spearman correlation analysis was conducted between 29 metabolites from key metabolic pathways and 15 differentially abundant microbial genera (Fig. 5a and b). Except for *Nanosyncoccus* and *Enterocloster*, which showed no significant correlations with any metabolites, all other genera in the microbiota exhibited significant positive or negative correlations with various metabolites (*P* < 0.05).

### Targeted metabolite validation between normal and abnormal group

Significant correlations were observed between TCA cycle-related metabolites and specific gut microbiota, suggesting that alterations in microbial abundance may regulate the biosynthesis of key serum metabolites. This dysregulation of the TCA cycle likely leads to systemic metabolic disequilibrium, ultimately contributing to the development of skeletal abnormalities in broilers.

We performed a targeted quantitative analysis of metabolites involved in energy metabolic pathways, specifically glycolysis and the TCA cycle. Significant differences were observed in the levels of FA, MA, and SA between the normal and abnormal groups (Fig. 5c, e–i). This consistent trend was observed in both the non-targeted and targeted metabolomic analyses. Furthermore, Spearman correlation analysis demonstrated that FA, MA, and SA exhibited positive or significantly positive correlations with the bone formation markers PINP, BALP, and COL1A1, while showing negative or significantly negative correlations with the bone resorption markers TRAP and β-CTX (Fig. 5d).

Six machine learning models were employed to evaluate and compare biomarker classification performance. The results demonstrated that FA and MA exhibited moderate to high diagnostic performance across all six models (0.5 < AUC ≤ 1) and showed good discriminative ability. Subsequently, ROC analysis was performed using FA and MA as a combined biomarker panels. The K-Nearest Neighbor and Logistic Regression models achieved perfect discrimination with AUC values of 1.00 (Fig. 5e–h), indicating their strong capability to discriminate the studied biomarkers.

### Effects of L-malic acid on geometric properties of the tibia and humerus

After supplementation with different levels of L-MA, there were significant differences in bone tibial weight, bone length, Pew, WTv, CSMI, and CSA among different treatment groups (*P* < 0.05), and bone length, Pew, CSMI, and CSA showed significant quadratic effects (*P* < 0.05). Moreover, the MMA group showed higher values or values that were significantly higher than the other treatment groups. Significant differences were observed in humeral bone weight, bone length, and Pew among different treatment groups (*P* < 0.05), whereas no significant differences were found in the remaining indices (Table 3).

**Table 3:** Effects of exogenous L-malic acid supplementation on the bone geometric parameters of the tibia and humerus

Items			Treatment^1^				SEM	*P*-value^2^		
			CON	LMA	MMA	HMA		Treat	Linear	Quadratic
Tibia	Weight, g		4.94^a^	4.93^a^	5.36^b^	5.03^a^	0.06	0.032	0.222	0.196
	Length, mm		68.52^a^	68.44^a^	70.27^b^	66.99^a^	0.33	0.002	0.346	0.026
	Pew, mm		17.07^ab^	17.30^b^	17.58^b^	16.49^a^	0.14	0.035	0.250	0.027
	Dew, mm		13.21	12.02	13.41	12.68	0.26	0.229	0.933	0.904
	M-L	Hh, mm	4.95^b^	4.96^b^	5.34^c^	4.60^a^	0.06	0.000	0.219	0.003
		hh, mm	2.30	2.38	2.39	2.15	0.08	0.686	0.530	0.488
	A-P	Hv, mm	4.46^ab^	4.69^bc^	4.85^c^	4.30^a^	0.06	0.005	0.533	0.003
		hv, mm	2.08	2.37	2.03	2.07	0.05	0.077	0.459	0.371
	MRWT		1.22	1.12	1.34	1.23	0.06	0.675	0.650	0.903
	WTv, mm		1.19^a^	1.16^a^	1.41^b^	1.11^a^	0.04	0.020	0.992	0.221
	CI		0.54	0.52	0.55	0.53	0.02	0.895	0.884	0.990
	CIv		0.54	0.49	0.58	0.51	0.01	0.057	0.827	0.898
	CSMI, mm^4^		20.70^ab^	24.20^bc^	29.15^c^	17.73^a^	1.21	0.003	0.721	0.005
	CSA, mm^2^		13.57^a^	13.82^a^	16.52^b^	12.09^a^	0.43	0.001	0.654	0.017
Humerus	Weight, g		2.79^b^	2.81^b^	2.84^b^	2.49^a^	0.05	0.018	0.029	0.010
	Length, mm		47.89^b^	47.37^ab^	48.56^b^	46.46^a^	0.23	0.006	0.134	0.068
	Pew, mm		14.91^ab^	14.77^a^	15.47^b^	14.32^a^	0.12	0.003	0.321	0.056
	Dew, mm		12.01	12.12	14.47	11.75	0.11	0.107	0.656	0.130
	M-L	Hh, mm	4.97	4.84	4.86	4.57	0.08	0.355	0.102	0.241
		hh, mm	1.94	2.20	2.06	2.10	0.07	0.650	0.607	0.655
	A-P	Hv, mm	4.06	4.25	4.41	4.00	0.06	0.090	0.971	0.064
		hv, mm	2.00	2.12	2.03	1.93	0.06	0.758	0.571	0.591
	MRWT		1.48	1.24	1.34	1.17	0.09	0.626	0.281	0.555
	WTv, mm		1.03	1.06	1.19	1.03	0.03	0.181	0.581	0.257
	CI		0.60	0.55	0.58	0.54	0.02	0.563	0.324	0.605
	CIv		0.51	0.50	0.54	0.52	0.01	0.721	0.578	0.888
	CSMI, mm^4^		16.11	17.86	19.84	13.80	0.93	0.124	0.560	0.092
	CSA, mm^2^		12.88	12.34	13.53	11.18	0.37	0.145	0.245	0.246

^a–c^ Means within a row with no common superscript differ significantly (*P* < 0.05)

^1^Different supplementary level (CON, control group; LMA, 0.05% L-MA; MMA, 0.15% L-MA; HMA, 0.3% L-MA)

^2^Treat, Linear and Quadratic represent Treat, linear and quadratic effects of L-MA addition

### Effects of L-malic acid on calcium and phosphorus concentrations in serum and tibia

Dietary supplementation with different levels of L-MA significantly increased serum Ca concentrations (*P* < 0.05), but significantly decreased serum P concentrations and the [Ca × P] product (*P* < 0.05). Supplementation with different doses of L-MA increased tibial Ca and P contents, with the significant increase observed in the MMA group (*P* < 0.05). These parameters exhibited significant linear and quadratic effects (*P* < 0.05; Table 4).

**Table 4:** Effects of exogenous L-malic acid on the serum calcium, phosphorus and bone composition

Items		Treatment^1^				SEM	*P-*value^2^		
		CON	LMA	MMA	HMA		Treat	Linear	Quadratic
Serum	Ca, mg/dL	5.62^a^	6.32^b^	6.08^b^	6.24^b^	0.07	< 0.001	0.004	0.001
	P, mg/dL	16.32^b^	11.95^a^	11.34^a^	10.56^a^	0.55	< 0.001	< 0.001	< 0.001
	[Ca × P], mg^2^/dL^2^	92.21^b^	95.27^a^	68.89^a^	66.12^a^	3.09	0.008	0.001	0.003
Tibia	Ash, %	40.77	40.81	44.56	42.15	0.65	0.118	0.008	0.026
	Ca, %	13.63^a^	14.17^a^	15.52^b^	14.46^a^	0.21	0.004	0.039	0.012
	P, %	6.11^a^	6.43^ab^	6.91^c^	6.64^bc^	0.13	0.003	0.006	0.003

^a–c^ Means within a row with no common superscript differ significantly (*P* < 0.05)

^1^ Different supplementary level (CON, control group; LMA, 0.05% L-MA; MMA, 0.15% L-MA; HMA, 0.3% L-MA)

^2^ Treat, Linear and Quadratic represent Treat, linear and quadratic effects of L-MA addition

### Effects of L-malic acid on bone histomorphology, metabolic markers, and gene expression

**Fig. 6:**
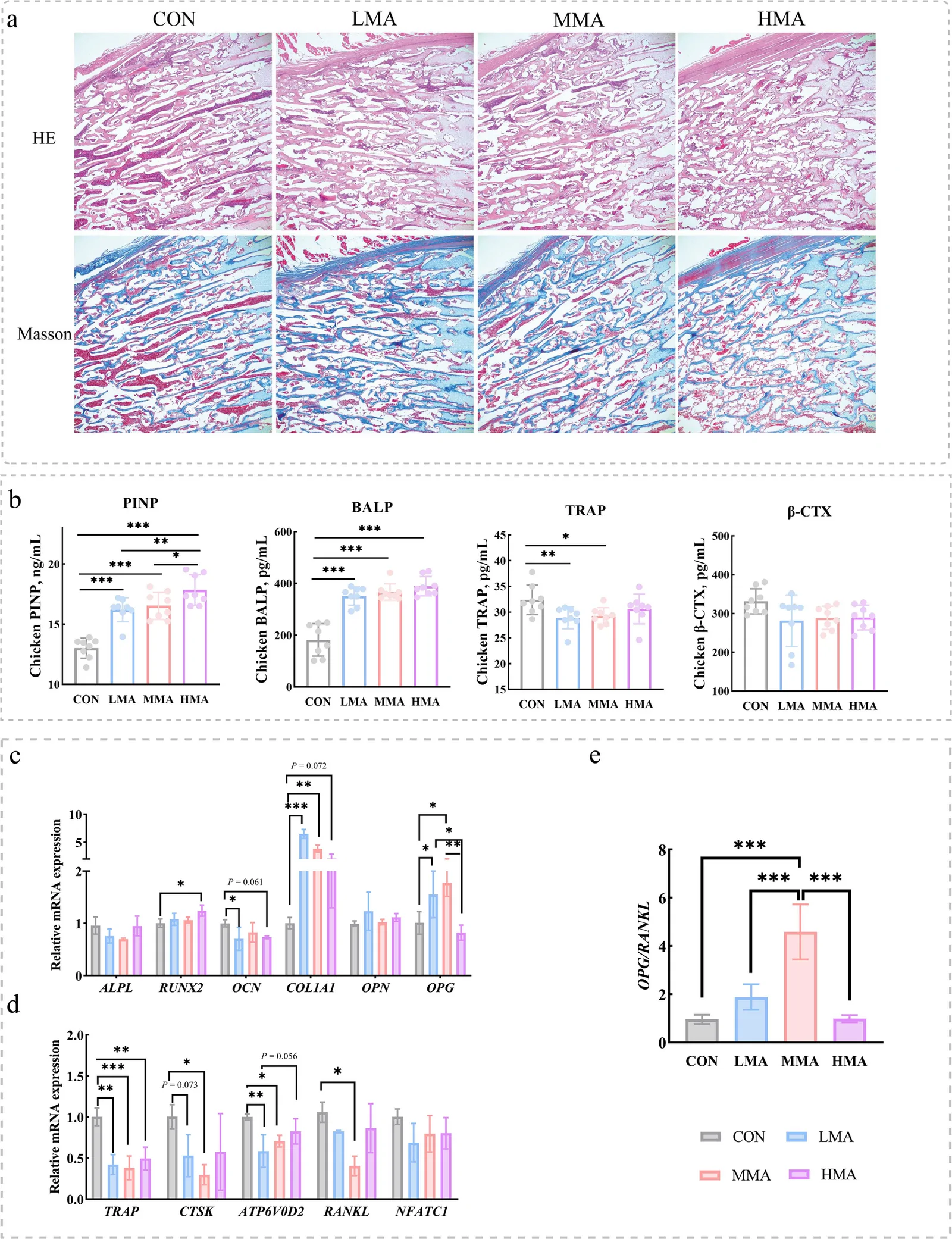
Effects of L‑malic acid on bone histomorphology, serum bone metabolism markers, and mRNA expression of bone formation and resorption related genes in broilers. **a** H&E and Masson staining of different treatment groups (scale bar, 200 μm). **b** Serum levels of bone formation (PINP, BALP) and bone resorption (TRAP, β-CTX) markers. **c** mRNA expression of bone formation related gene. **d** mRNA expression of bone resorption related gene. **e** The OPG/RANKL ratio. ^*^P < 0.05; ^**^P < 0.01; ^***^P < 0.001

Compared with the CON group, trabecular bone was denser and exhibited greater trabecular interconnectivity following L-MA supplementation. The blue‑stained regions in the Masson’s stain images indicate newly formed bone. These images show that the bone tissue revealed by H&E staining almost completely overlaps with the newly formed bone tissue in the Masson stain, indicating active bone growth and development at d 21 (Fig. 6a).

The results for serum bone metabolism markers showed that supplementation with different levels of L-MA significantly increased PINP and BALP concentrations (*P* < 0.05). Compared with the CON group, the LMA and MMA groups showed significantly reduced serum TRAP concentrations (Fig. 6b).

The mRNA expression levels of bone formation and bone resorption related genes in bone tissue are shown in Fig. 6c–e. The results indicated that supplementation with medium and high levels of L-MA significantly increased the expression levels of *COL1A1* and *OPG* (*P* < 0.05). Furthermore, the HMA group showed a significant increase in *RUNX2* mRNA expression (*P* < 0.05). Supplementation with different levels of L-MA reduced the expression levels of bone resorption related genes to varying degrees. In particular, the MMA group significantly reduced the mRNA expression levels of *TRAP*, *CTSK*, *ATP6V0D2*, and *RANKL* (*P* < 0.05).

Furthermore, the OPG/RANKL ratio in the MMA group was significantly higher than in the other treatment groups (*P* < 0.05).

### Effects of L-malic acid on bone microstructural parameters and bone formation proteins

Based on the comprehensive analysis of skeletal geometric properties, Ca and P metabolism, and the mRNA expression levels of bone resorption and formation related genes, medium-dose L-MA supplementation was preliminarily identified as the most effective for improving bone health in this study. Therefore, the CON group and the MMA group were selected for further analysis.

**Fig. 7:**
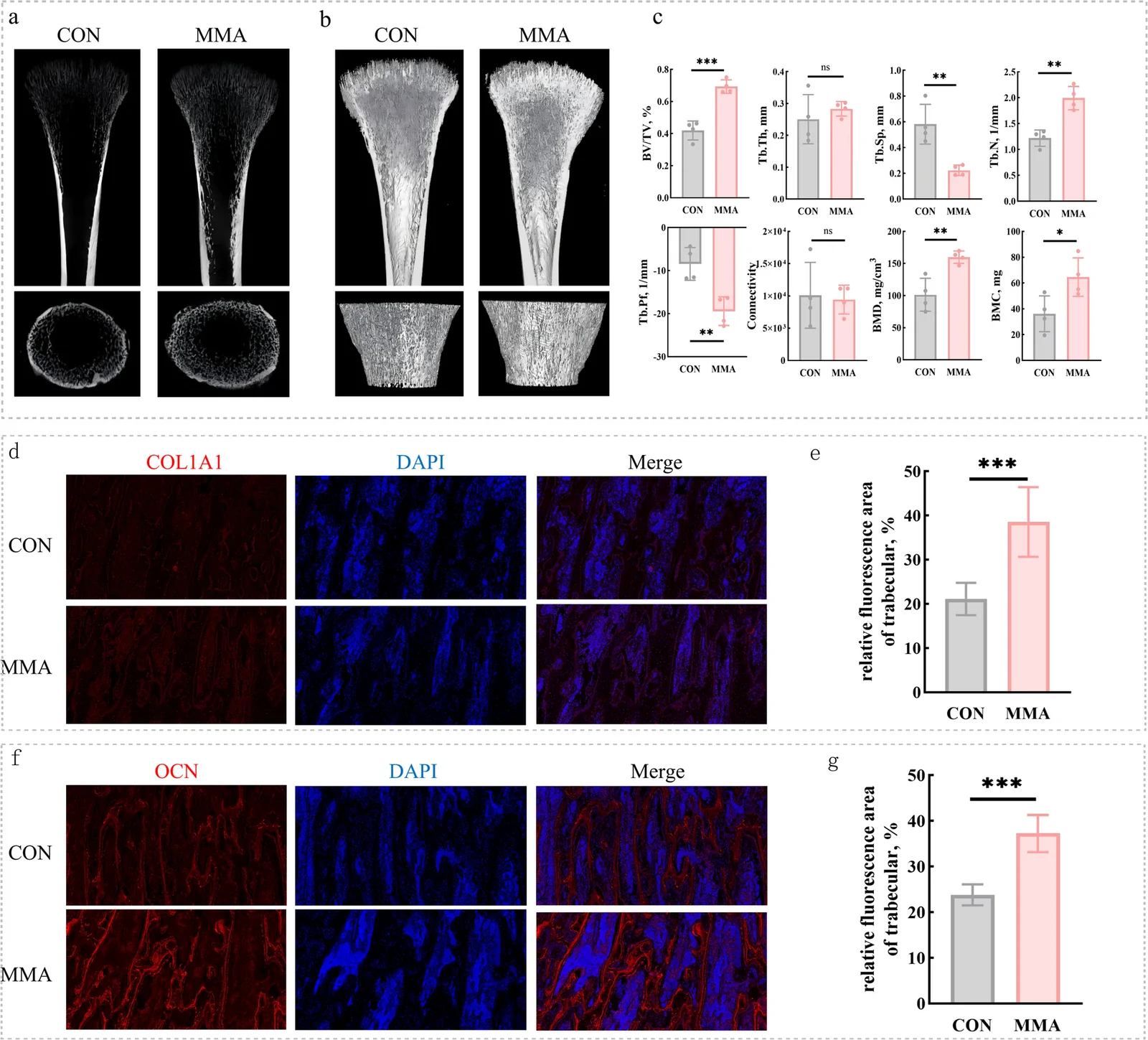
Effects of L‑malic acid on tibial bone microstructure and bone formation protein expression. **a** Two-dimensional micro-CT images of the proximal tibia in the CON group and the MMA group (Scale bar, 10 mm). **b** Three-dimensional micro-CT images of the proximal tibia in the CON group and the MMA group (Scale bar, 10 mm). **c** Quantitative analysis of trabecular bone microstructural parameters. **d** and **e** Representative images of COL1A1 immunofluorescence staining (**d**) and quantitative analysis of panel d (**e**). **f** and **g** Representative images of OCN immunofluorescence staining (**f**) and quantitative analysis of panel f (**g**) (Scale bar, 100 μm). ^*^P < 0.05; ^**^P < 0.01; ^***^P < 0.001; ns, not significant

The results showed that supplementation with medium-dose L-MA significantly increased BV/TV, Tb.N, BMD, and BMC, while significantly decreasing Tb.Sp and Tb.Pf (*P* < 0.05; Fig. 7a–c).

Immunofluorescence staining for COL1A1 and OCN was performed to analyze the distribution of bone formation-related proteins following L-MA supplementation. The results showed that the expression of COL1A1 and OCN on the trabecular bone in the MMA group was significantly higher than that in the CON group (*P* < 0.05; Fig. 6d–g). These findings indicate that supplementation with medium-dose L-MA promotes the expression of the bone formation-related proteins COL1A1 and OCN, increases the number of trabeculae, and reduces trabecular separation.

### Effects of L-malic acid on energy metabolites and mitochondrial function

**Fig. 8:**
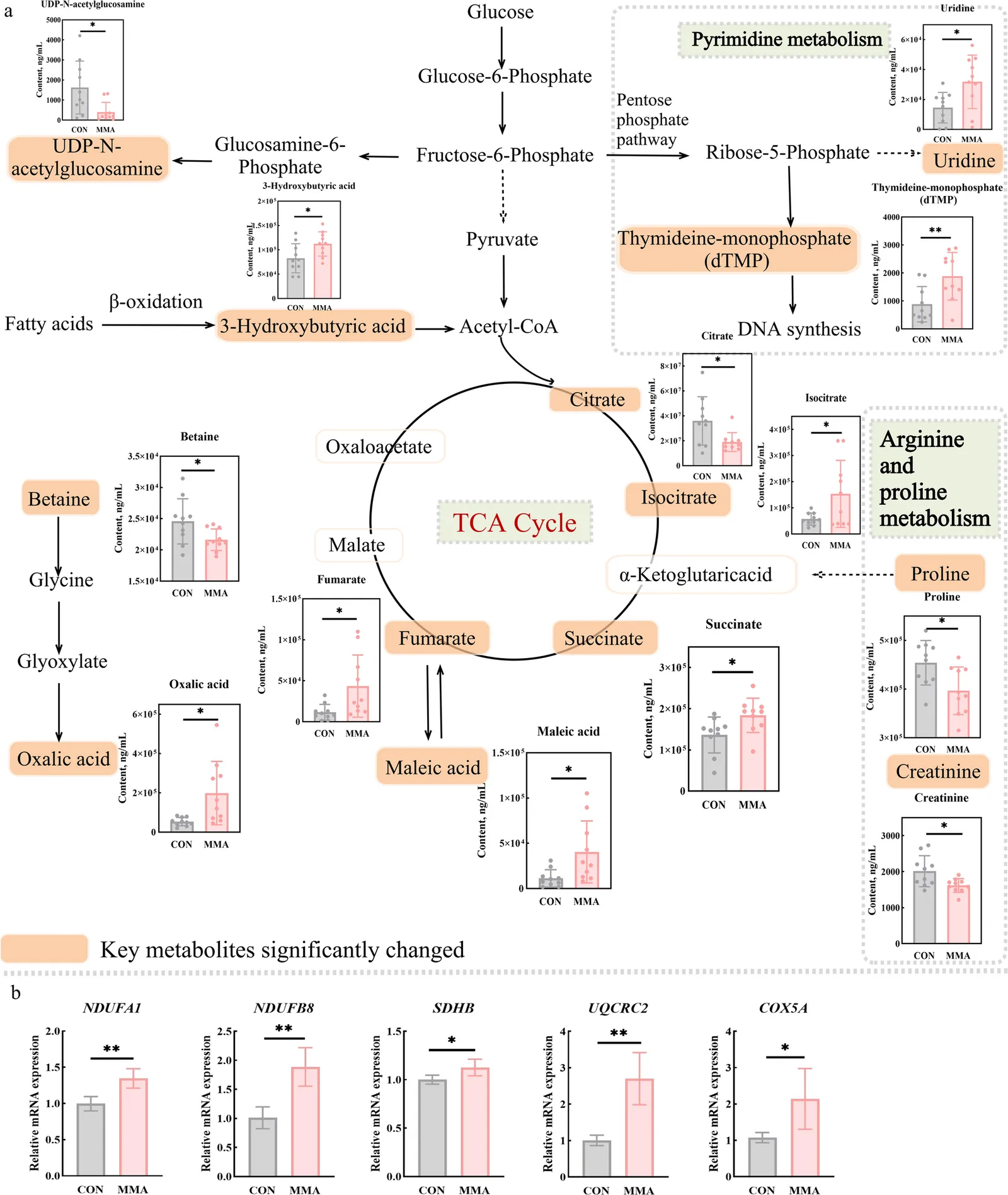
Effects of L‑malic acid on serum metabolic pathways and mRNA expression of electron transport chain complexes in bone tissue. **a** Differential analysis of metabolic pathways between the CON group and the MMA group. **b** Analysis of mRNA expression levels of electron transport chain complexes in the CON group and the MMA group. ^*^P < 0.05; ^**^P < 0.01

Targeted analysis of serum energy metabolites was performed in the CON and MMA groups. The results showed significant differences in 13 metabolites, including thymidine monophosphate (dTMP), oxalic acid, UDP-N-acetylglucosamine, proline, creatinine, uridine, maleic acid, fumarate, citrate, succinate, 3-hydroxybutyric acid, isocitrate and betaine (*P* < 0.05; Fig. 8a). KEGG enrichment analysis revealed significant differences in 16 metabolic pathways, including the citrate cycle (TCA cycle), Oxidative phosphorylation (*P* < 0.05; Fig. S3b).

Specifically, the levels of dTMP and uridine in the pyrimidine metabolism pathway, maleic acid, fumarate, succinate and isocitrate in the TCA cycle; and 3-hydroxybutyric acid and oxalic acid were significantly elevated in the MMA group (*P* < 0.05). Conversely, the levels of UDP-N-acetylglucosamine, citrate, betaine, proline and creatinine were significantly reduced in the MMA group (*P* < 0.05). The Fig. 8b shows that the mRNA levels of *SDHB*, *NDUFB8*, *UQCRC2*, and *COX5A* were significantly higher in the MMA group than in the CON group (*P* < 0.05).

## Discussion

Modern advances in intensive broiler farming have led to improved management conditions and superior feed quality; however, the accompanying skeletal issues cannot be overlooked. Excessive loading negatively affects bone growth and development, leading to deterioration of bone structure and mechanical properties [54]. Bone geometric parameters determine the biological function and mechanical properties of bone [55]. Ca and P, essential nutrients for skeletal growth, are deposited in the bone tissue in the form of hydroxyapatite under certain conditions [56]. Ca and P are delivered to the epiphyseal growth plate to support endochondral bone mineralization and skeletal maturation. Insufficient mineralization may result in skeletal disorders in broilers [57]. Key indicators for assessing bone health include fundamental bone characteristics, geometric properties, trabecular microstructural parameters, and bone mineral density. Geometric bone parameters significantly influence the skeletal function and biomechanical competence [55]. In the present study, the reduction in geometric properties of the tibia and humerus in the abnormal group, particularly evident in the tibia, along with deterioration of microstructural parameters in the proximal tibia (e.g., BMD, Tb.N and Tb.Sp) and lower bone Ca and P contents, collectively highlight the severity of skeletal impairment present in broiler production.

Bone homeostasis during broiler development is maintained by the dynamic balance between osteoblast-mediated bone formation and osteoclast-mediated bone resorption [58]. To decipher the relationship between skeletal abnormalities and bone metabolism, we measured serum concentrations of the bone formation markers BALP, PINP, and COL1A1 and bone resorption markers β-CTX and TRAP. Compared with the normal group, the abnormal group exhibited decreased serum levels of osteogenic markers and increased levels of osteoclastic markers, indicating that skeletal abnormalities in cage-reared broilers may result from enhanced bone resorption, which disrupts bone tissue homeostasis.

Skeletal disorders are closely associated with osteoblasts or osteoclasts [59–61]. Osteoblasts play a critical role in bone formation, a process comprising three stages: proliferation, differentiation, and mineralization. RUNX2 serves as a crucial transcription factor during osteoblast proliferation and maturation, thereby promoting osteoblast differentiation [61]. BALP, a matrix protein expressed during matrix maturation, is an early biochemical marker of osteogenesis [62, 63]. Following matrix mineralization, late-stage osteogenic markers such as OPN and OCN are expressed. Finally, the deposition of hydroxyapatite into the bone matrix requires interaction with COL1A1 to form mineralized collagen that is essential for maintaining bone strength [63], PINP reflects the synthesis rate of type I collagen and the bone turnover status [64]. In the present study, the abnormal group showed reduced serum levels of BALP, PINP, and COL1A1, as well as decreased mRNA expression of *RUNX2*, *ALPL*, *OCN*, and *COL1A1* in the bone tissue compared with the normal group. Collectively, these findings suggest impaired osteoblast differentiation and reduced osteogenic function in the abnormal group.

Osteoclasts are specialized multinucleated cells primarily responsible for bone resorption [65]. The primary osteoclastogenic signal, receptor activator of nuclear factor-κB ligand (RANKL), binds to its receptor, RANK, and subsequently activates NFATC1, the master transcription factor for osteoclastogenesis [66–68]. This transcription factor stimulates osteoclast differentiation. Once differentiated, osteoclasts adhere tightly to the bone surface, forming a ruffled border that increases the contact area with the bone [69]. ATP6V0D2, a component of V-ATPase, serves as a specific proton pump that is essential for extracellular acidification during bone resorption [70]. Simultaneously, NFATC1 translocates to the nucleus and activates the expression of genes encoding acid hydrolases, such as *TRAP*, *CTSK*, and *MMP9* [71], which collectively mediate bone demineralization and degradation of organic bone matrix components [72–75]. Elevated β-CTX levels indicate an increased rate of matrix degradation, reflecting enhanced bone resorption [74]. In the present study, the abnormal group exhibited significantly higher serum levels of TRAP and β-CTX, along with elevated mRNA expression of *TRAP*,* CTSK*, and *MMP9* in bone tissue, compared with the normal group. ROS have been established as key signaling molecules in osteoclast differentiation, activation and bone resorptive function. Studies have demonstrated that ROS generated downstream of the RANKL/RANK signaling pathway promote osteoclastogenesis, cell fusion, and bone resorptive activity [76]. In the present study, ROS levels were significantly elevated in bone tissue of the abnormal group. These results indicate an enhanced osteoclastic bone resorption state in the abnormal group. Combined with the observed phenotypic differences, the skeletal abnormalities in the abnormal group may be attributed to increased osteoclast formation, enhanced activity, and consequently, elevated bone resorption capacity.

As specialized bone-resorbing cells in bone tissue, osteoclasts require a substantial energy supply to support their growth and differentiation, as well as to fuel the secretion of acids and proteolytic enzymes [77]. Glycolysis and mitochondrial energetics represent the two primary metabolic pathways that convert nutrients into ATP, thereby providing energy for fundamental biological processes. OXPHOS serves as the principal bioenergetic mechanism in osteoclastogenesis, operating in conjunction with glycolysis [78–80]. For instance, Deng et al. [80] demonstrated that mitochondrial respiration is critical for upregulating CTSK expression, whereas Rohatgi et al. [81] reported that epigenetic factors can regulate osteoclast activity by modulating mitochondrial respiration. In the present study, serum metabolomics analysis revealed that the differentially enriched pathways between the abnormal and normal groups were primarily involved in energy metabolism, including the TCA cycle, amino acid metabolism, and OXPHOS. Based on these findings, we hypothesized that the increased osteoclast activity in broilers with skeletal abnormalities may be attributed to alterations in their energy metabolism patterns. Furthermore, bone tissue ATP and ROS levels were significantly increased in the abnormal group, accompanied by significantly higher mRNA expression of key glycolytic enzymes and key mitochondrial ETC-related genes compared with the normal group. This indicates a change in the metabolic profile of the abnormal group, which may be associated with enhanced osteoclast differentiation. This observation aligns with the findings of Czupalla et al. [82], who reported increased expression of metabolic enzymes associated with the citric acid cycle and OXPHOS during osteoclast differentiation. Furthermore, several studies have shown that RANKL guides metabolic reprogramming toward OXPHOS during osteoclast differentiation through multiple regulatory mechanisms [83–87]. Therefore, OXPHOS is a critical bioenergetic pathway that supports osteoclast differentiation. Li et al. [78] demonstrated that deletion of the glucose transporter gene *Glut1* in osteoclast progenitors reduced aerobic glycolysis but did not impair OXPHOS; however, it still attenuated osteoclast differentiation in vitro. Consistent results have been observed in human [88] and chicken derived cells [86], confirming that glucose is the primary energy substrate for osteoclast differentiation and function. In addition to fueling OXPHOS, aerobic glycolysis also increases during osteoclast differentiation [77, 78, 86].

In the present study, the depletion of TCA cycle intermediates (fumarate and malate) co-occurred with the synchronous upregulation of glycolysis and OXPHOS genes. This seemingly paradoxical phenomenon may reflect an adaptive strategy of osteoclasts under energy stress: TCA cycle suppression and glycolysis and OXPHOS compensation. Indeed, decreased concentrations of these metabolites may also reflect an accelerated TCA cycle flux. Alternatively, osteoclasts in the abnormal group could have sacrificed TCA cycle intermediates to fuel the high energy demands of bone resorption by enhancing glycolysis and OXPHOS, rather than these changes resulting from reduced TCA cycle activity. This parallels the depletion of TCA cycle intermediates observed during prolonged exercise and muscle fatigue [88–90], highlighting the dynamic regulation of the metabolic pool under high energy demands. This challenges the traditional view that mitochondrial activation is synonymous with the accumulation of TCA cycle metabolites.

**Fig. 9:**
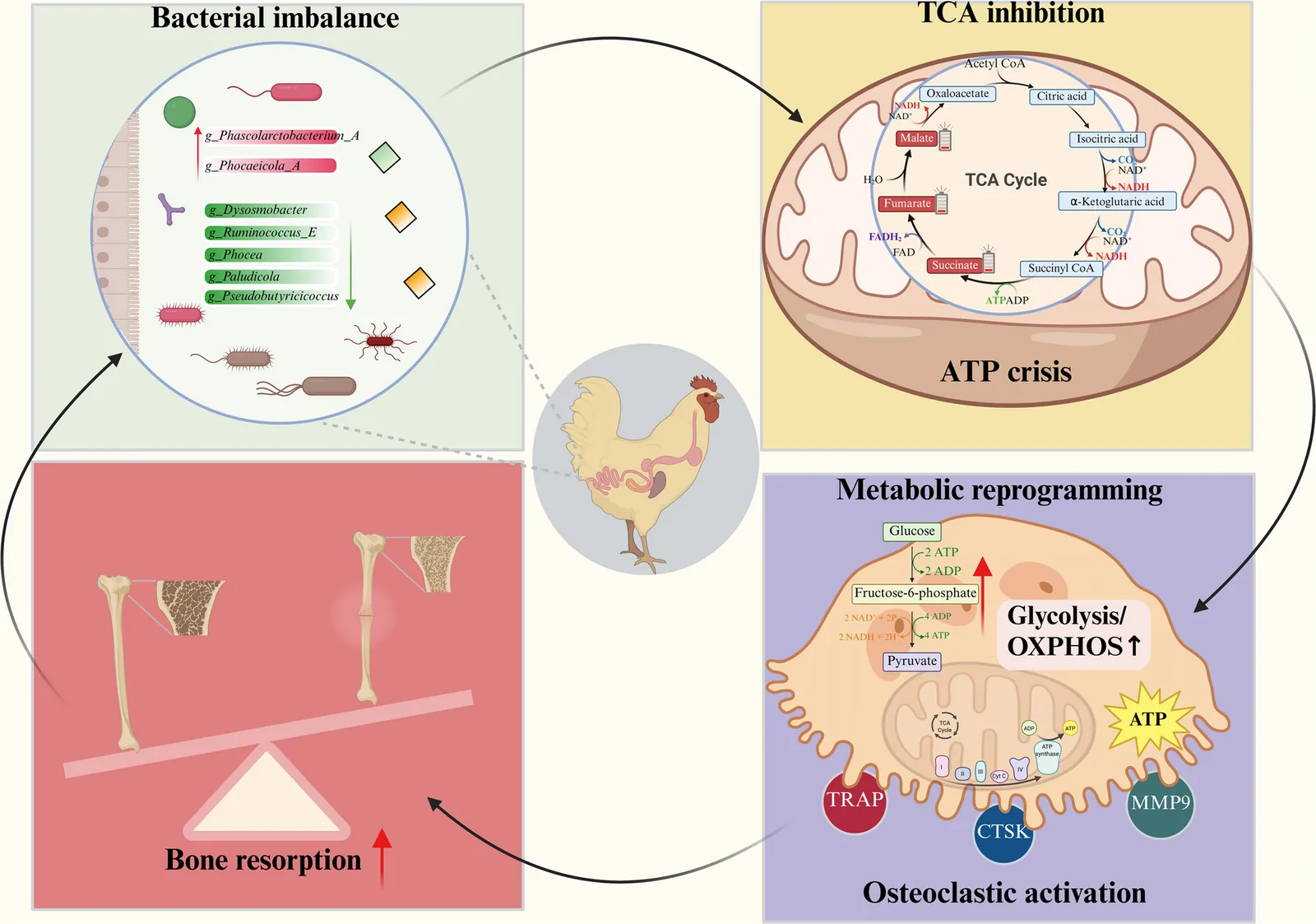
Schematic illustration of the main findings in broilers with skeletal abnormalities. Created in Biorender (https://BioRender.com/d511ina)

Targeted metabolomics validation confirmed that the levels of FA, MA, and SA were markedly lower in the abnormal group. TCA cycle metabolites exert positive effects on osteogenesis and promote bone regeneration, remodeling, and repair [91]. Similarly, Liu et al. [92] demonstrated that supplementation with TCA metabolite-mimetic hydrogels promotes bone tissue regeneration. In the present study, depletion of the TCA metabolite pool, particularly the reduced levels of succinate, fumarate, and malate, inhibited osteogenic differentiation, impaired bone formation and compromised skeletal homeostasis. To meet the high bioenergetic demands of osteoclastic bone resorption, glycolysis and OXPHOS are compensatorily activated, further exacerbating the consumption of the TCA metabolite pool and ultimately leading to disrupted bone homeostasis (Fig. 9).

Serum metabolite profiles are closely linked to gut microbiota composition and microbial metabolic activity. Nutrients absorbed through gut microbial metabolic processes enter the systemic circulation and subsequently exert systemic regulatory effects on target organs, including bone, via the gut–bone axis [93, 94]. The gut microbiota play a pivotal role in bone metabolism [95, 96]. In this study, the results revealed a significant increase in Firmicutes_C and its representative genus *Phascolarctobacterium_A* in the abnormal group. *Phascolarctobacterium_A* is a Gram-negative, non-spore-forming, strict anaerobe recognized as a primary consumer of succinate [97]. Succinate metabolism can reduce host TCA cycle activity [98]. An increased abundance of *Phascolarctobacterium_A* has been reported in the gut microbiota of humans and animal models of osteoporosis [96, 99, 100]. Zang et al. investigated the associations between gut microbiota and 15 different diseases and identified 14 genera that were consistently enriched in diseased individuals, including *Phascolarctobacterium_A* [101]. *Phocaeicola_A* was initially classified as *Bacteroides*, but is now one of the dominant genera within Bacteroidota [102]. Recently, several members of the genus *Bacteroides* were reclassified as *Phocaeicola* [102, 103]. *Phocaeicola_A* alters mitochondrial function and disrupts normal energy metabolism [104]. In the present study, the abundance of these microbial taxa was significantly elevated in the abnormal group. Hierarchical clustering indicated a close correlation between *Phocaeicola_A* and *Phascolarctobacterium_A*, suggesting their potential synergistic role in influencing host energy metabolism and skeletal health.

*Dysosmobacter*, a host-beneficial commensal bacterium [105], has the capacity to produce succinate and contribute to microbial energy metabolism [106]. *Ruminococcus_E* is a butyrate-producing bacterium, and *Pseudobutyricicoccus* belongs to the family Butyricicoccaceae; both are generally recognized as beneficial short-chain fatty acid (SCFA)-producing taxa [107]. Butyrate serves as a substrate for the TCA cycle, and its deficiency can lead to impaired mitochondrial energy metabolism and reduced synthesis of TCA cycle intermediates such as malate and fumarate [108]. *Phocea* and *Paludicola* belong to the family Ruminococcaceae, which is a group of important commensal bacteria capable of degrading cellulose and polysaccharides to produce SCFAs [109]. Families Butyricicoccaceae and Ruminococcaceae are phylogenetically related families that are considered beneficial microbial taxa; however, their abundances were significantly reduced in the abnormal group. These significantly depleted microorganisms in the abnormal group belonged to the phylum Firmicutes_A, class Clostridia, and order Oscillospirales. A positive association has been reported between the Oscillospirales and health, highlighting its potential as a source of next-generation probiotic candidates [110, 111]. The SCFAs produced by these beneficial bacteria play a vital role in bone homeostasis. As precursors to TCA cycle-related metabolites, SCFAs positively influence osteogenic differentiation and suppress bone resorption [112, 113]. The reduced abundance of these microbial taxa in the abnormal group suggests a potential adverse effect on bone homeostasis.

The microorganisms that were significantly reduced in the abnormal group exhibited positive or significantly positive correlations with depleted metabolites, particularly TCA cycle intermediates. These findings suggest a potential link between microbiota, metabolites, and skeletal metabolic reprogramming in broilers with skeletal abnormalities, and indicate that microbial dysbiosis is associated with the depletion of TCA cycle intermediates. This finding supports the interpretation that the decrease in TCA intermediates in the abnormal group was not due to increased metabolic flux but rather to gut microbiota dysbiosis-associated depletion of these key metabolites. To meet the high bioenergetic demands of osteoclasts, compensatory upregulation of glycolysis-and mitochondrial ETC-related genes occurs (Fig. 9). This aligns with the compensation–repression model proposed by the Marian Walhout research group, which posits that when a metabolic pathway is impaired, organisms upregulate other genes within the same reaction pathway to maintain metabolic function [114].

Notably, the mechanistic evidence provided in this study relies primarily on mRNA expression and metabolomics data, representing a significant limitation. Future investigations are warranted to incorporate protein‑level validation, mitochondrial function assessments, and functional osteoclast assays to provide a more comprehensive understanding of the involvement of TCA cycle dysfunction in broiler skeletal disorders.

Based on the identification of FA and MA as biomarkers associated with skeletal abnormalities in broilers through multi‑omics analysis, and considering the green, safe, and sustainable characteristics of L‑MA as a dietary supplement, as well as its in vivo interconversion with fumaric acid, L‑MA was selected for further evaluation of its effects on bone health in yellow‑feathered broilers. The results showed that L‑MA supplementation improved bone health, as evidenced by increased tibial Ca and P deposition, enhanced bone mechanical properties, elevated serum levels of bone formation markers, and upregulated expression of osteogenesis-related genes, and an increased OPG/RANKL ratio.

Importantly, targeted metabolomics analysis revealed that the levels of TCA cycle intermediates were significantly increased in the L-MA treatment group, and the mRNA expression levels of mitochondrial ETC complexes were also markedly upregulated. These results indicate that exogenous L-MA can enhance TCA cycle activity and mitochondrial respiratory capacity. These findings are consistent with those of Wu et al. [115], who demonstrated that exogenous L-MA increases TCA cycle intermediates, accelerates cellular bioenergetic metabolism, and promotes biosynthesis. Tong et al. [116] also reported that L-MA enhances TCA cycle activity by upregulating the expression of key enzymes of the TCA cycle. Moreover, several studies have shown that various exogenous additives promote bone formation and mineralization precisely through the regulation of TCA cycle metabolism [117, 118]. Based on the findings of the present study, it is speculated that the beneficial effect of L‑MA on bone health may be related to its promotion of energy metabolism, enhancement of the energy supply to osteoblast-lineage cells, and subsequent facilitation of bone matrix synthesis and mineralization.

Notably, in the present study, L-MA supplementation significantly increased serum Ca levels but decreased serum P levels. Given the marked deposition of P in bone, we hypothesized that L-MA may accelerate the uptake of circulating P and its subsequent deposition into bone tissue. Studies have shown that P ions play a more critical role than Ca ions during the early phase of mineralization [119, 120]. Mineralization is not a simple equimolar deposition of Ca and P; rather, it is initiated by the combination of phosphate and calcium ions within matrix vesicles secreted by osteoblasts, leading to the formation of amorphous calcium phosphate, which subsequently transforms into hydroxyapatite crystals. Therefore, after L-MA supplementation, serum phosphorus is rapidly transported to bone tissue and participates in nucleation for mineralization, leading to a transient decrease in serum phosphorus levels. Nevertheless, the dynamic changes in Ca and P metabolism induced by L-MA warrant further investigation.

Therefore, L-MA not only serves as an important metabolic biomarker for identifying skeletal abnormalities, but also represents a promising functional additive for improving locomotor disorders in broilers. However, the present study primarily validated metabolic alterations at the individual and tissue levels. Whether L-MA indirectly affects bone metabolism through modulation of the gut microbiota warrants further investigation.

## Conclusions

This study indicates that skeletal abnormalities in yellow-feathered broilers are characterized not only by insufficient bone mineralization, reduced skeletal mechanical integrity, and restricted bone formation but are also closely associated with disturbances in energy metabolism; impaired TCA cycle activity likely represents an important metabolic basis underlying their occurrence and progression. As a key metabolite closely related to skeletal abnormalities, L-MA not only has potential value as a metabolic biomarker but also has the potential to improve bone health by enhancing TCA cycle activity, thereby promoting Ca and P deposition and extracellular bone matrix mineralization. These findings provide new evidence for understanding the metabolic regulatory mechanisms underlying skeletal abnormalities in poultry and offer a theoretical basis for the development of bone health-targeted nutritional strategies based on functional metabolites.

## Supplementary Information

Additional file 1: Table S1. Ingredients and nutrient levels of basic diets by stage (air-dry basis, %). Table S2. The primers used for qPCR assays. Table S3. Microbial abundance at the phylum level in normal and abnormal groups (Top10). Table S4. Microbial abundance at the genus level in normal and abnormal groups (Top25). Fig. S1. Permutation tests of the OPLS-DA models in positive (a) and negative (b) ion modes. Fig. S2. Gut microbiota composition in normal and abnormal groups. Fig. S3. KEGG enrichment analysis of targeted metabolomics.

## Data Availability

The raw 16S rRNA gene sequencing data reported in this paper are available from the National Centre for Biotechnology Information (NCBI) with the Sequence Read Archive (SRA) number PRJNA1348693.

## Abbreviations

β-CTX: Beta-isomerized C-terminal telopeptide of type I collagen
ALPL: Alkaline phosphatase
ATP: Adenosine triphosphate
BALP: Bone alkaline phosphatase
COL1A1: Collagen type I
COX5A: Cytochrome c oxidase subunit 5A (complex IV)
CTSK: Tissue proteinase K
ENO1: Enolase 1
HK2: Hexokinase 2
LDHA: Lactate dehydrogenase A
NDUFB8: NADH:ubiquinone oxidoreductase subunit B8 (complex I)
OCN: Osteocalcin
OPN: Osteopontin
PFKM: Phosphofructokinase muscle type
PKLR: Pyruvate kinase liver and red blood cells
RANKL: Receptor activator of nuclear factor-κB ligand
ROS: Reactive oxygen species
RUNX2: Runt-associated transcription factor 2
SDHB: Succinate dehydrogenase complex iron sulfur subunit B (complex II)
TRAP: Tartrate-resistant acid phosphatase
UQCRC2: Ubiquinol-cytochrome c reductase core 2 (complex III)
