# Supplementary material for: Integrated metabolomic and gut microbiota analyses reveal TCA cycle dysfunction in broiler skeletal disorders and the protective effect of L‑malic acid
Source: J Anim Sci Biotechnol. 2026 Sep 13;17:182. doi: 10.1186/s40104-026-01498-5 (PMC13571327; doi:10.1186/s40104-026-01498-5)
Supplement: Supplementary file 1 [file 40104_2026_1498_MOESM1_ESM.docx]

Integrated metabolomic and gut microbiota analyses reveal TCA cycle dysfunction in broiler skeletal disorders and the protective effect of L‑malic acid

Yuyang Xue ^1^, Min Yao ^2^, Xiangjian Peng ^1^, Fang Wang ^1^, Wei Jing ^1^, Fei Zhang ^1^, and Lulu Mi ^1^, Wenju Zhang ^1^, Cunxi Nie ^1 *^

^1^ College of Animal Science and Technology, Bingtuan Key Laboratory for Efficient Utilization of Non-Grain Feed Resources, Shihezi University, Shihezi, China

^2^ School of Medicine, Shihezi University, Shihezi, China

*Corresponding author.

Email address: [niecunxi@shzu.edu.cn](mailto:niecunxi@shzu.edu.cn)

Supplementary Table S1

Table S1. Ingredients and nutrient levels of basic diets by stage (air-dry basis, %)

| Items | Content | | |
| --- | --- | --- | --- |
|  | 1 to 21 days | 22 to 42 days | 43 to 63 days |
| **Ingredients，%** | | | |
| Corn | 60.08 | 58.98 | 56.54 |
| Soybean meal | 9.50 | — | — |
| Cottonseed meal | 10.00 | 10.00 | 7.00 |
| Wheat middling | — | 5.00 | 10.00 |
| Barley grain | 5.00 | 5.00 | 5.00 |
| Corn gluten meal | 5.50 | 3.50 | 3.00 |
| Corn distillers dried grains with solubles | 3.00 | 3.00 | 3.00 |
| Corn gluten meal | — | 4.50 | 4.50 |
| Feather meal, hydrolyzed | 1.00 | 1.50 | 2.00 |
| Meat and bone meal | — | 3.00 | 3.00 |
| Lard | 1.00 | 2.00 | 3.00 |
| Premix^1^ | 4.92 | 3.52 | 2.96 |
| **Total** | 100.00 | 100.00 | 100.00 |
| **Nutrient levels^2^，%** | | | |
| ME/(MJ/kg) | 12.35 | 12.77 | 13.19 |
| CP | 19.55 | 17.61 | 16.48 |
| Ca | 0.93 | 0.81 | 0.75 |
| TP | 0.59 | 0.50 | 0.47 |
| Lys | 1.08 | 0.98 | 0.88 |
| Met+Cys | 0.76 | 0.71 | 0.65 |

^1^The premix provided the following per kg of diet. 1 - 21 d: vitamin A 12 000 IU, vitamin D_3_ 600 IU, vitamin E 45 IU, vitamin K 2.5 mg, vitamin B_1_ 2.4 mg, vitamin B_2_ 5.0 mg, vitamin B_6_ 2.8 mg, vitamin B_12_ 16 µg, nicotinic acid 42 mg, pantothenic acid 12 mg, folic acid 1.0 mg, biotin 0.12 mg, choline 1 300 mg, Cu 7 mg, Fe 80 mg, 80 mg, I 0.7 mg, Mn 60 mg, Se 0.15 mg. 22 -42 d: vitamin A 9 000 IU, vitamin D_3_ 500 IU, vitamin E 35 IU, vitamin K 2.2 mg, vitamin B_1_ 2.3 mg, vitamin B_2_ 5.0 mg, vitamin B_6_ 2.4 mg, vitamin B_12_ 15 µg, nicotinic acid 35 mg, pantothenic acid 10 mg, folic acid 0.7 mg, biotin 0.1 mg, choline 1 000 mg, Cu 7 mg, Fe 80 mg, Zn 60 mg, I 0.6mg, Mn 60 mg, Se 0.15 mg. 43 – 63 d, vitamin A 9 000 IU, vitamin D_3_ 500 IU, vitamin E 35 IU, vitamin K 2.2 mg, vitamin B_1_ 2.3 mg, vitamin B_2_ 5.0 mg, vitamin B_6_ 2.4 mg, vitamin B_12_ 15 µg, nicotinic acid 35 mg, pantothenic acid 10 mg, folic acid 0.7 mg, biotin 0.1 mg, choline 1 000 mg, Cu 7 mg, Fe 80 mg, Zn 60 mg, I 0.6mg, Mn 60 mg, Se 0.15 mg.

^2^ Metabolizable energy, Lys and Met + Cys are calculated; other values are measured

Table S2 The primers used for qRT-PCR assays

| **Gene name** | **Primer sequence (5′→3′)** | **Accession** |
| --- | --- | --- |
| *β-actin* | F: TATGTGCAAGGCCGGTTTC  R: TGTCTTTCTGGCCCATACCAA | NM_205518.2 |
| *Runx2* | F: CACGCTGCTAAACCCAAACT  R: GACTCATCCATCCTGCCACT | NM_204128.2 |
| *OCN* | F: GAAGAGGCAGAAGAGGTTCG  R: AGATAGTCACAGGGAGGGTAGC | NM_001201386.2 |
| *OPN* | F: TAGGAGTTGCTGCTGGGATT | NM_204535.5 |
|  | R: CCTGGTGGTACCTGTGTGTG |  |
| *COL1A1* | F: TTGACCCTAACCAAGGATGC | [XM_054359445.1](https://www.ncbi.nlm.nih.gov/entrez/viewer.fcgi?db=nucleotide&id=2462617021) |
|  | R: CACCCCTTCTGCGTTGTATT |  |
| *ALPL* | F: GGAGAAGGACCCCGAATACTG  R: TTGACGCCGCAGAGGTAAG | NM_205360.2 |
| *OPG* | F: CCTACTCGTTTCACACCCA | XM_015283019.2 |
|  | R: TTGTTTTGCTGCTTCCATAA |  |
| *Nfatc1* | F: TGCAGCGCTGTATAGTGTGT | XM_047430218.1 |
|  | R: TGTGTTACAGTTAATGAGCCACC |  |
| *ATP6V0D2* | F: ATGTGGACCACGGCTACTTG | NM_001008455.2 |
|  | R: GCTATGGGTCGCTGGTGTAA |  |
| *MMP9* | F: GCCATCACTGAGATCAATGGAG | NM_204667.2 |
|  | R: GATAGAGAAGGCGCCCTGAGT |  |
| *TRAP* | F: CTGGCTTTGGGCGATAACT  R: TCGGAGTGTCGGCTGTATG | XM_040693093.2 |
| *Cts K* | F: ACGTCCCGGAGGTTGATTTG | NM_204971.3 |
|  | R: CCACCTCCTCGCTGGTCATA |  |
| *HK2* | F: GCAGAAGGTGGAGATGGAGAG | XM_046903343.1 |
|  | R: CGGGAAGGAGAAGGTGAAGC |  |
| *PFKM* | F: GATATGGTTGGCGGCTGGAC | XM_046904942.1 |
|  | R: GCTCCTCAAACTTGGCTCTCC |  |
| *PKLR* | F: TTGAAGTGTGTAGTTGTTTCCAGAC | XM_046924612.1 |
|  | R: CAGCAGAACCGTTACCATAAGC |  |
| *LDHA* | F: TCATCAAACTGAAGGGTTACACATC | NM_205284.2 |
|  | R: CACAAGGAACACTTAGGAAGACATC |  |
| *ENO1* | F: TGGAAATGGATGGAACGGAGAAC | XM_046931163.1 |
|  | R: AGCAGGAACAGGCAGAATGAC |  |
| *UQCRC2* | F: CTGCCCATCTGCTTCGTCTC | XM_424611.7 |
|  | R: GGAGCCGTGGTGACATTAAGG |  |
| *NDUFB8* | F: CTGTGGGACCGAAGCAGTATC | NM_001006502.2 |
|  | R: GTGAAAACTAAAGAAGATGCCAACC |  |
| *NDUFA1* | F: AACGGCGGCAAGGAGAAGAG | NM_001302115.2 |
|  | R: ATCTGTGGGTTCTTTGAGGTGTG |  |
| *SDHB* | F: GACTTGAGTAACTTCTACGCACAG | NM_001278039.2 |
|  | R: TTGTCCCCATTCCACCAGTAAC |  |
| *COX5A* | F: CGTATTTCAACAAACCAGACATCG | XM_003641804.6 |
|  | R: TTCACCACCTCCAGAATGCG |  |

F forward primer, R reverse primer

*Runx2*, runt-related transcription factor 2; *OCN*, osteocalcin; *OPN*, osteopontin; *COL1A1*, collagen type I alpha 1 chain; *ALPL*, alkaline phosphatase; *OPG*, osteoprotegerin; *Nfatc1,* nuclear factor of activated T-cells 1; *ATP6V0D2*，ATPase H+ transporting V0 subunit d2；*MMP9,* matrix metallopeptidase 9*; TRAP*, tartrate-resistant acid phosphatase; *Cts K*, cathepsin K; *HK2,* hexokinase 2*; PFKM,* phosphofructokinase*; PKLR,* pyruvate kinase*; LDHA,* lactate dehydrogenase A*; ENO1*, enolase 1*; UQCRC2*, ubiquinol-cytochrome c reductase core protein II*; NDUFB8*, ubiquinone oxidoreductase subunit B8; *NDUFA1,* ubiquinone oxidoreductase subunit A1; *SDHB*, succinate dehydrogenase complex iron sulfur subunit B; *COX5A*, cytochrome c oxidase subunit 5A.

Tablle S3 Microbial abundance at the phylum level in Normal and Abnormal groups (Top10)

| Item | Normal | Abnormal | SEM | *P*-value |
| --- | --- | --- | --- | --- |
| Firmicutes_A | 54.66 | 48.10 | 4.34 | 0.148 |
| Bacteroidota | 26.92 | 30.60 | 4.21 | 0.394 |
| Actinobacteriota | 5.79 | 5.67 | 1.92 | 0.950 |
| Firmicutes_C | 3.60 | 5.89 | 0.74 | 0.006 |
| Firmicutes_D | 4.85 | 4.32 | 1.56 | 0.735 |
| Proteobacteria | 1.62 | 1.94 | 0.76 | 0.682 |
| Firmicutes_B | 1.30 | 1.71 | 0.28 | 0.168 |
| Patescibacteria | 0.251 | 0.761 | 0.26 | 0.064 |
| unclassified_Bacteria | 0.38 | 0.46 | 0.10 | 0.456 |
| Desulfobacterota_I | 0.38 | 0.39 | 0.11 | 0.894 |

Table S4 Microbial abundance at the genus level in Normal and Abnormal groups (Top25)

| Item | Normal | Abnormal | SEM | *P*-value |
| --- | --- | --- | --- | --- |
| *Alistipes_A* | 14.73 | 17.73 | 2.55 | 0.255 |
| *Faecalibacterium* | 9.09 | 6.42 | 1.47 | 0.086 |
| *Parabacteroides_B* | 4.37 | 4.01 | 1.25 | 0.773 |
| *Blautia_A* | 4.20 | 4.35 | 0.83 | 0.860 |
| *unclassified_Lachnospiraceae* | 4.03 | 3.35 | 0.57 | 0.246 |
| *Barnesiella* | 3.70 | 1.81 | 2.16 | 0.394 |
| *Mediterraneibacter_A* | 3.42 | 4.16 | 0.70 | 0.298 |
| *Thermophilibacter* | 3.31 | 3.49 | 1.88 | 0.926 |
| *Phascolarctobacterium_A* | 3.10 | 4.89 | 0.75 | 0.029 |
| *Faecousia* | 2.46 | 1.86 | 0.51 | 0.255 |
| *Gemmiger_A* | 2.23 | 2.45 | 0.39 | 0.577 |
| *Lawsonibacter* | 2.21 | 1.62 | 0.32 | 0.082 |
| *CAG-269* | 1.69 | 1.40 | 0.63 | 0.651 |
| *Negativibacillus* | 1.46 | 1.28 | 0.24 | 0.460 |
| *Enterenecus* | 1.29 | 1.39 | 0.75 | 0.903 |
| *Lactobacillus* | 1.21 | 0.33 | 0.42 | 0.052 |
| *CAG-273* | 1.18 | 1.45 | 0.43 | 0.547 |
| *Aphodousia* | 1.18 | 0.69 | 0.31 | 0.143 |
| *unclassified_Oscillospiraceae* | 1.16 | 0.75 | 0.19 | 0.045 |
| *Peptococcus* | 1.14 | 1.49 | 0.27 | 0.209 |
| *Phocaeicola_A* | 1.11 | 3.67 | 0.91 | 0.012 |
| *Bacteroides_H* | 1.05 | 1.40 | 0.27 | 0.212 |
| *Collinsella* | 1.01 | 0.39 | 0.37 | 0.107 |
| *Ligilactobacillus* | 1.00 | 1.06 | 0.79 | 0.938 |
| *Limosilactobacillus* | 0.78 | 1.42 | 0.51 | 0.221 |

Fig S1. Permutation tests of the OPLS-DA models in positive (a) and negative (b) ion modes. The x-axis represents the similarity with the original model, and the y-axis shows the R² and Q² values. The validation parameters of the OPLS‑DA model in negative mode were R^2^X(cum) = 0.273, R^2^Y(cum) = 0.997, and Q^2^(cum) = 0.593. The positive ion mode yielded validation parameters of R^2^X(cum) = 0.284, R^2^Y(cum) = 0.996, and Q^2^(cum) = 0.656. Q^2^ values exceeding 0.5 indicated good predictive ability of the modelsAll permuted Q² values were lower than the original Q² values, and the Q² intercepts were close to zero, confirming that the models were not overfitted and exhibited good predictive ability.

Fig S2. Gut microbiota composition in Normal and Abnormal groups. (a) Venn diagram of ASV/OTU abundance differences in cecal microbiota between the Normal and Abnormal groups. (b) Alpha-diversity analysis between groups assessed by the Simpson and Pielou_e indices. (c) Composition analysis of cecal microbiota at the genus level in the Normal and Abnormal groups. (d) LEfSe analysis between the Normal and Abnormal groups.

Fig S3. KEGG enrichment analysis of targeted metabolomics. (a) KEGG pathway analysis based on the targeted metabolic profiles of the Normal and Abnormal groups. (b) KEGG pathway analysis based on the targeted metabolic profiles of the CON and MMA groups.
